# Fine mapping of *qAHPS07* and functional studies of *AhRUVBL2* controlling pod size in peanut (*Arachis hypogaea* L.)

**DOI:** 10.1111/pbi.14076

**Published:** 2023-05-31

**Authors:** Hui Yang, Lu Luo, Yuying Li, Huadong Li, Xiurong Zhang, Kun Zhang, Suqing Zhu, Xuanlin Li, Yingjie Li, Yongshan Wan, Fengzhen Liu

**Affiliations:** ^1^ State Key Laboratory of Crop Biology, Shandong Key Laboratory of Crop Biology College of Agronomy, Shandong Agricultural University Tai'an China

**Keywords:** peanut, pod size, fine mapping, *qAHPS07*, *AhRUVBL2*

## Abstract

Cultivated peanut (*Arachis hypogaea* L.) is an important oil and cash crop. Pod size is one of the major traits determining yield and commodity characteristic of peanut. Fine mapping of quantitative trait locus (QTL) and identification of candidate genes associated with pod size are essential for genetic improvement and molecular breeding of peanut varieties. In this study, a major QTL related to pod size, *qAHPS07*, was fine mapped to a 36.46 kb interval on chromosome A07 using F_2_, recombinant inbred line (RIL) and secondary F_2_ populations. *qAHPS07* explained 38.6%, 23.35%, 37.48%, 25.94% of the phenotypic variation for single pod weight (SPW), pod length (PL), pod width (PW) and pod shell thickness (PST), respectively. Whole genome resequencing and gene expression analysis revealed that a RuvB‐like 2 protein coding gene *AhRUVBL2* was the most likely candidate for *qAHPS07*. Overexpression of *AhRUVBL2* in *Arabidopsis* led to larger seeds and plants than the wild type. *AhRUVBL2*‐silenced peanut seedlings represented small leaves and shorter main stems. Three haplotypes were identified according to three SNPs in the promoter of *AhRUVBL2* among 119 peanut accessions. Among them, SPW, PW and PST of accessions carrying *Hap_ATT* represent 17.6%, 11.2% and 26.3% higher than those carrying *Hap_GAC*，respectively. In addition, a functional marker of *AhRUVBL2* was developed. Taken together, our study identified a key functional gene of peanut pod size, which provides new insights into peanut pod size regulation mechanism and offers practicable markers for the genetic improvement of pod size‐related traits in peanut breeding.

## Introduction

The cultivated peanut (*Arachis hypogaea* L.) is an important oil and cash crop widely grown in the world. In 2019, peanut cultivation covered an area of 29.6 million ha with a total production of approximately 48.8 million tons (http://www.fao.org/faostat/en/#data/QC). Peanut is a rich source of nutrients and healthy bioactive compounds for human consumption, including high‐quality oils, proteins, resveratrol, isoflavones and phytic acid (Bertioli *et al*., 2016). In recent years, edible peanut oil and food have been increasingly consumed globally, therefore, the improvement of pod yield has become the primary goal in peanut breeding. The pod yield per unit area of peanut is determined by a combination of three elements: single pod weight (SPW), the number of pods per plant, and the number of plants per area (Gomes and Lopez, 2005). Peanut pod size is significantly correlated with SPW, and it is generally accepted that pod size is one of the important factors influencing peanut pod yield (Chen *et al*., 2017; Chu *et al*., 2020; Fonceka *et al*., 2012; Khedikar *et al*., 2017; Luo *et al*., 2017, 2018). Therefore, exploring the genetic and molecular mechanisms of pod size regulation has a wide prospect on molecular breeding of peanut.

Peanut is a leguminous plant that produces flowers aerially and develops pods underground. The pod development can be divided into three stages: pod expansion, seed filling and desiccation. The increase of pod size mainly occurs in the pod expansion stage, after which it remains almost the same (Chen *et al*., 2016b). The pod shell provides sufficient space for seed development (Clevenger *et al*., 2016). Previous research has indicated that pod shell thickness (PST) is significantly correlated with pod size (Ignacio *et al*., 1981). In general, the final size of fruit or seed is coordinately controlled by cell number and/or cell size (Orozco‐Arroyo *et al*., 2015). Grain size of rice is controlled by cell proliferation and cell expansion in the spikelet hull (Li *et al*., 2018), and silique length and grain weight are affected by the size of the silique wall cells in rapeseed (Liu *et al*., 2015). For peanut, recent studies with Zhonghua16, Yuanza9102 and their smaller pod mutants indicated that the size of the pod shell cells determined the overall pod size (Wan *et al*., 2017; Wang *et al*., 2022). However, the developmental mechanisms regulating pod size are complex and remain poorly understood.

The identification of QTLs for pod size‐related traits is an important means to elucidate the genetic basis of pod size. In the past 10 years, traditional QTL analysis of biparental populations of peanut has been frequently employed to identify genomic regions and closely linked molecular markers for pod size‐related traits. A number of QTLs have been identified for pod length (PL), pod width (PW) and pod weight. These QTLs are usually pleiotropic or show an aggregated distribution (Chen *et al*., 2016a, 2017; Fonceka *et al*., 2012; Gangurde *et al*., 2020; Huang *et al*., 2015; Khedikar *et al*., 2017; Luo *et al*., 2017, 2018). For instance, two major QTLs for hundred‐pod weight (HPW), three QTLs for PL and six major QTLs for PW were mapped in a BC_2_F_2:3_ population. Of these, two major QTLs for HPW and two major QTLs for PW were distributed in the same marker intervals on chromosomes B02 and B05 (Fonceka *et al*., 2012). These studies indicate that linkage analysis can effectively locate QTLs and analyse the genetic relationships of pod size‐related traits. However, developing high‐density whole genome linkage maps for peanut is an expensive process.

With the application of next‐generation sequencing technologies (NGS) in peanut (Varshney *et al*., 2014a, 2014b) and the publication of genomic sequencing of the wild and tetraploid cultivated peanut (Bertioli *et al*., 2016, 2019; Chen *et al*., 2019; Yin *et al*., 2018; Zhuang *et al*., 2019), the development of polymorphism molecular marker studies and QTL mapping has become more convenient. A simple and efficient approach‐ BSA‐seq, based on the integration of bulked segregant analysis (BSA) and NGS, has been successfully used for candidate genomic region prediction in crops. This approach has also been successfully used in mapping the target interval for pod and seed‐related traits in groundnut, including testa colour (Yuan *et al*., 2013), shelling percentage (SP) (Luo *et al*., 2019a), seed size (Zhuang *et al*., 2019) and fresh seed dormancy (Kumar *et al*., 2020). These studies indicated that BSA‐seq can provide the target interval for pod‐related traits, but it is difficult to assess the genetic efficacy of QTLs. The strategy of combining BSA‐seq with linkage mapping in the target QTL region has been proposed, which combines the advantages of the two QTL mapping methods and has already been applied to identify QTLs related to seed size and red testa in peanut (Zhang *et al*., 2022a; Zhuang *et al*., 2019).

The large size of the peanut genome and genetic complexity of pod size‐related traits make positional cloning of pod size regulatory genes difficult and no related genes have been reported so far. Because of their similar genetic background, secondary mapping populations have been successfully applied to the fine mapping of QTLs and identification of candidate genes for grain‐related traits in other crops, such as soybean (He *et al*., 2014b), rice (Zhu *et al*., 2019), rapeseed (Wang *et al*., 2020b), maize (Chen *et al*., 2020a), wheat (Chen *et al*., 2020b) and barley (Watt *et al*., 2020). In peanut, a population of chromosome segment substitution lines (CSSL) was used to shorten QTLs interval for seed size from 5 Mb to 168.37 kb, which greatly improved the accuracy of QTL mapping (Alyr *et al*., 2020). The development of a secondary mapping population for peanut pod‐related traits is of great significance for fine mapping of QTLs and gene cloning.

The purpose of the present study was to fine map major QTL associated with pod size, clone key genes, develop tightly linked molecular markers for genetic improvement of pod size‐related traits, and provide theoretical support for regulatory mechanism studies of peanut pod size.

## Results

### Phenotypic analyses of the two parents, RIL and F_2_ populations

**Figure 1:**
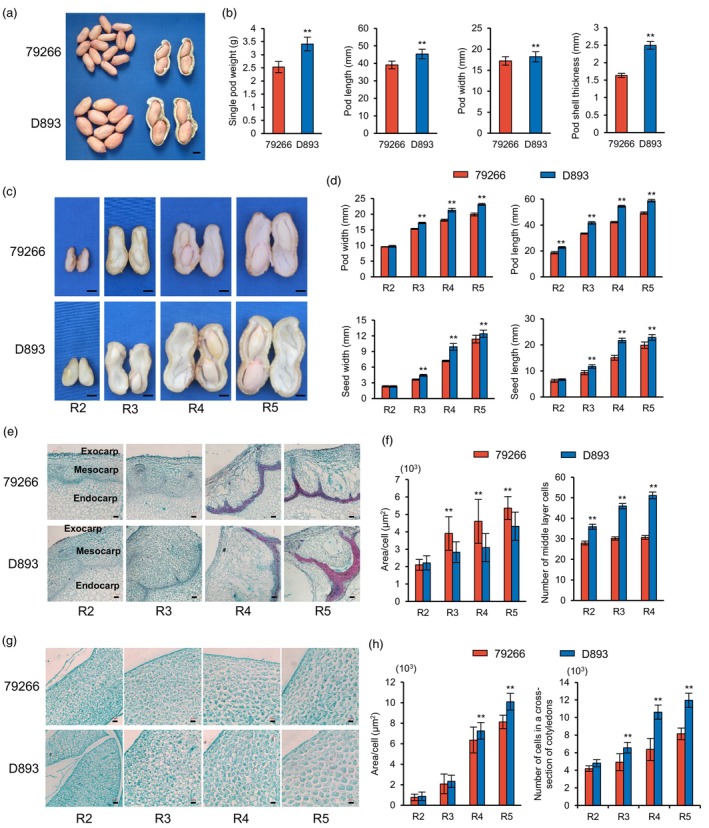
Phenotypic and cytological analysis of 79266 and D893 pods. (a) Phenotypes of mature dry pods from 79266 and D893. Bar = 1 cm. (b) Statistical data of single pod weight, pod length, pod width and pod shell thickness of mature dry pod from 79266 and D893. (c) Phenotypic characterizations of 79266 and D893 pods at R2–R5 stages. Bar = 1 cm. (d) Statistical data of pod length, pod width, seed length and seed width of 79266 and D893 at R2‐R5 stages. (e) The transverse sections of pod shell of 79266 and D893 from stages R2–R5. Bar = 200 μm. (f) Statistical data of cell areas and layers of the exocarp and mesocarp transverse sections of pod shell in 79266 and D893 pods from stages R2‐R5. (g) The transverse sections of cotyledon in 79266 and D893 from stages R2–R5. Bar = 100 μm. (h) Statistical data of cell areas and numbers of the exocarp and mesocarp of cotyledon transverse sections of 79266 and D893 from stages R2–R5. (Student's *t*‐test; *P < 0.05; **P < 0.01).

The two parent varieties, 79266 and D893, showed significant differences in mature pod size. Compared with 79266, D893 had a significantly higher single pod weight (SPW), pod length (PL), pod width (PW) and pod shell thickness (PST) under all seven environments (Figure 1a,b). In addition, D893 also showed significantly larger PL, PW, seed length (SL) and seed width (SW) in the four stages of rapid pod expansion (Figure 1c,d). In order to investigate potential differences between 79266 and D893, cytological analysis of pod shell and seed at the four stages revealed that the pod shell of D893 contained a significantly higher number of cells than 79266 at the R2‐R4 stage, and the difference gradually increased with pod development (Figure 1e,f). The cell areas were similar between 79266 and D893 at the R2 stage, but significantly higher in 79266 from the R3 to R5 stage (Figure 1e,f). In the cotyledons, the number of cells in the cross section in D893 was significantly higher than in 79266 at the R3‐R5 stage, with significantly higher cell areas in the R4‐R5 stage. Nevertheless, the difference in cell numbers was greater than that of cell area in cotyledons (Figure 1g,h). Taken together, these results indicated that the number of pod shell cells was the main reason for the difference in pod size between 79266 and D893.

In order to analyse the genetic basis of pod size‐related traits, a F_2_ population containing 1020 individual plants and a RIL population containing 151 lines were derived from 79266 and D893. A wide range of phenotypic variations and transgressive segregation for SPW, PL, PW, and PST were observed in both populations, and the frequency distribution of these traits showed a continuous variation with normal distributions (Figures S1 and S2; Tables S1 and S2). Correlation analysis between these traits were estimated in the F_2_ and RIL populations. With the exception of PL and PST, all other traits showed significant positive associations with each other in the F_2_ population, whereas the best linear unbiased prediction (BLUP) values from the RIL population indicated significant positive associations between all pod size‐related pod traits (Tables S3 and S4). In both populations, SPW represented higher correction with all other three traits.

### Identification of *qAHPS07*

#### Identification of genomic region controlling pod size

**Figure 2:**
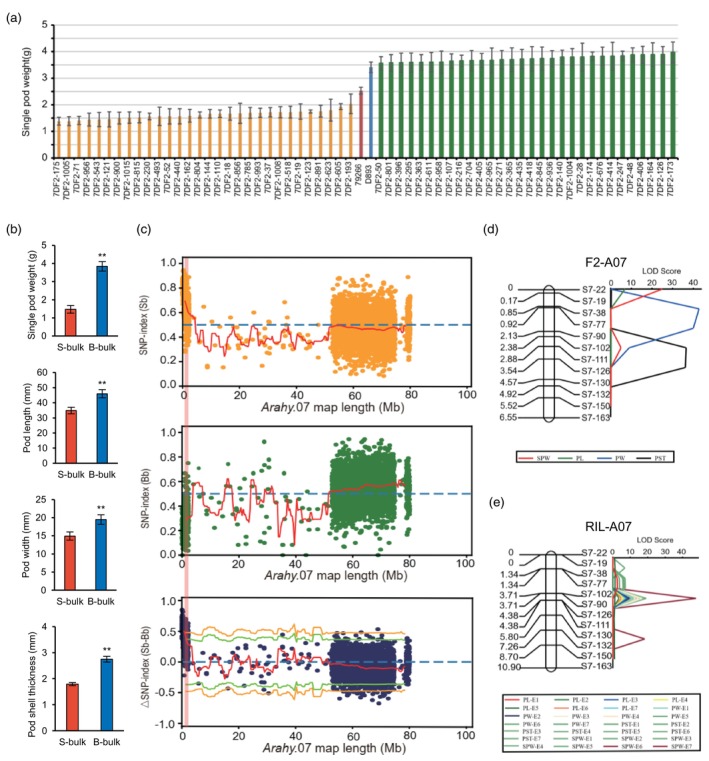
BSA‐seq and traditional mapping approach for mapping genomic regions controlling peanut pod size. (a) The statistical data of single pod weight of each F_2_ individual selected to construct the small and big bulks. (b) Pod weight, pod length, pod width and pod shell thickness of small (S) and big (B) bulks. (c) SNP index plot between S bulk and 79266 assembly (top), B bulk and 79266 assembly (middle) and ΔSNP index plot (bottom) of pseudomolecule A07 with statistical confidence interval under the null hypothesis of no QTLs (orange, *P* < 0.01; and green, *P* < 0.05). The significant genomic region identified for pod size‐related traits are shaded in pink (11.02–746.24 kb). (d) The linkage group created in the candidate region by BSA‐seq using F_2_ population (left), and the distribution of the LOD scores for pod size‐related traits (right). (e) The linkage group created in the candidate region by BSA‐seq using RIL population (left), and the distribution of the LOD scores for pod size‐related traits (right), LOD scores were calculated for the seven environments (E1, E2, E3, E4, E5, E6 and E7).

In order to map QTLs related to pod size, the BSA‐seq was performed using the F_2_ population. Therefore, SPW was taken as the reference standard, 30 F_2_ plants with high (3.56–3.97 g) and low (1.38–2.03 g) SPW were selected, respectively, to construct the big and small extreme bulks (Figure 2a). Significant differences were detected between pods form each bulk in SPW, PL, PW and PST (Figure 2b). 79266, D893 and two extreme bulks were re‐sequenced to map candidate genomic regions for pod size using BSA‐seq analysis (Table S5). Based on the SNP‐index and ΔSNP‐index plots, a 0.73 Mb (*Arahy*.07:11 016 ~ 746 246 bp) interval on chromosome A07 was identified as candidate genomic region for pod size (Figures 2c and [Link], [Link]).

#### QTL mapping for pod size‐related traits using F_2_ and RIL populations

To confirm the genomic regions and the genetic effects of QTLs for pod size‐related traits, linkage analysis was performed in the candidate genomic region using F_2_ and RIL populations. 12 KASP markers were selected to genotype the F_2_ and RIL populations (Table S6; Figure 2d,e). In the F_2_ population, two QTLs for SPW, one QTL for PL, one QTL for PW and one QTL for PST were detected in the interval from *S7‐19* to *S7‐126*, and these QTLs could explain 2.36%–19.97% of the phenotypic variation for four traits (Table S7; Figure 2d). In the RIL population, three QTLs for SPW, two QTLs for PL, two QTLs for PW and three QTLs for PST were detected in interval from *S7‐19* to *S7‐132*, and these QTLs could explain 8.47%–38.6% of the phenotypic variation for four traits (Table S8; Figure 2e).

Linkage analysis showed that there were pleiotropism gene or gene linkage associated with pod size between markers *S7‐19* and *S7‐132*, which could regulate several pod size‐related traits such as SPW, PL, PW and PST. This QTL cluster was named *qAHPS07*, and the physical location was narrowed in a 478.09 kb region on chromosome A07 (*Arahy*.07:56535 ~ 564268 bp).

### Fine mapping of the *qAHPS07*

**Figure 3:**
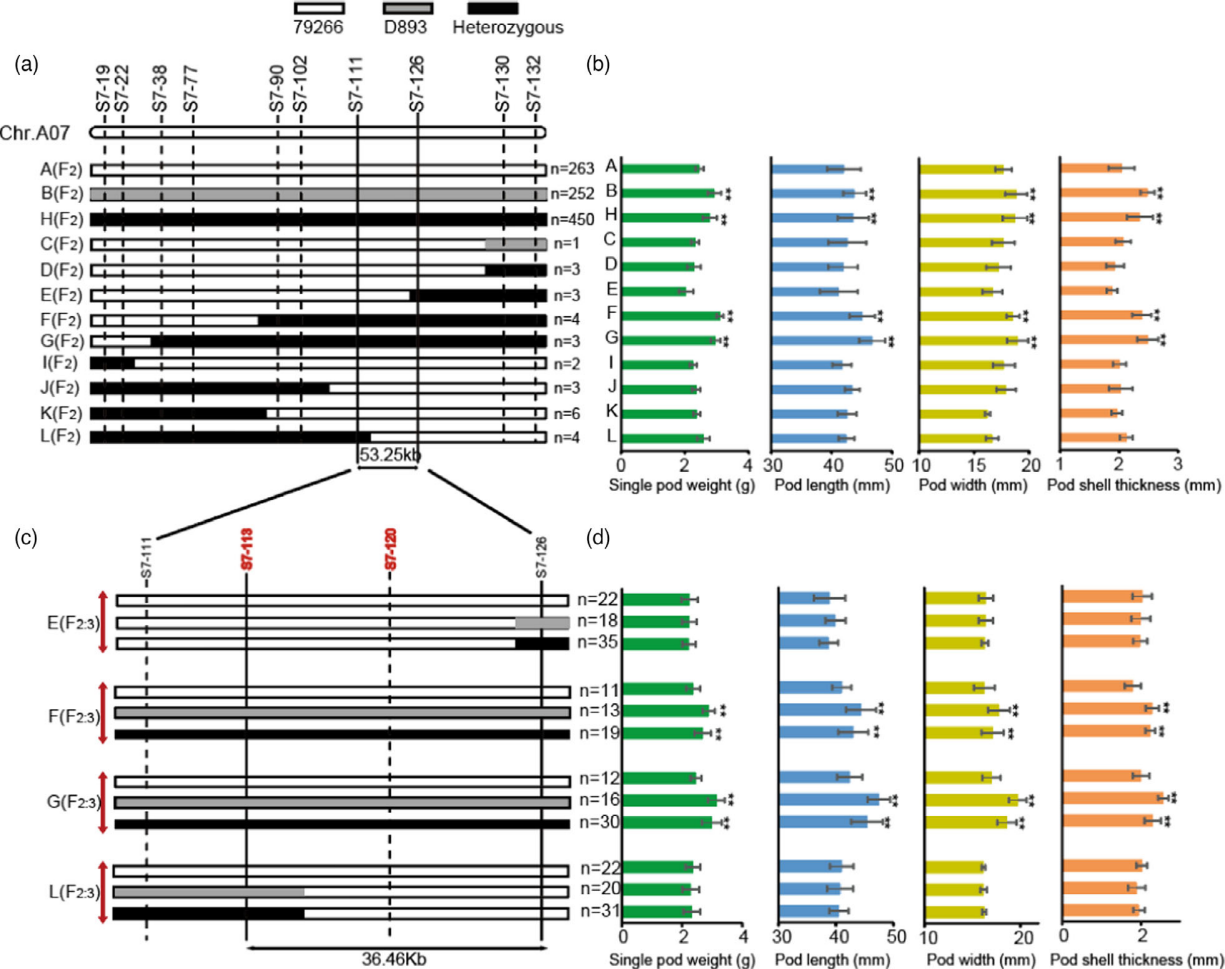
Fine mapping of the *qAHPS07* locus. (a) The graphical genotypes of the F_2_ recombinants and 12 markers used to screen secondary F_2_ individual plants. A, B and H represent the graphical genotypes of 79266 genotype, D893 genotype and heterozygous genotype, and C to L represent the graphical genotypes of 9 recombinants types. Arrows represent the 53.25 kb interval of fine mapping. ‘n’ represents the numbers of individual plants of each type. White, grey and black bars represent 79266, D893 and heterozygous haplotypes, respectively. (b) The statistical data of SPW, PL, PW and PST of each type. phenotypic comparisons of individual plants of each type for SPW, PL, PW and PST. (c) The graphical genotypes of the four F_2:3_ recombinants and the new markers used for further fine‐mapping of *qAHPS07* locus. (d) The statistical data of SPW, PL, PW and PST of each type. Significant differences are indicated by * (*P* < 0.05), **(*P* < 0.01) (Student's *t*‐test).

To fine‐map *qAHPS07*, a secondary F_2_ segregating population including 1039 individuals was developed by back‐crossing LA123 with 79266. LA123 was similar to D893 with super‐larger pods, and had a significantly higher SPW, PL, PW and PST than 79266. The results of genome re‐sequencing data of 79266, D893 and LA123 showed that LA123 was consistent with D893 in the initial mapping interval of *qAHPS07*, whereas in the remainder of the genome, the genomic similarity between LA123 and 79266 was 67.2% (Figure S6). 10 KASP markers were utilized to genotype and identify informative recombinants within the target region (Figure 3a). The 29 heterozygous recombinants were grouped into 9 recombination types (named as C‐L). Phenotypic analysis showed that, similar with D893, heterozygous genotype between *S7‐111* and *S7‐126* (recombination type H, F and G) lead to significantly higher SPW, PL, PW and PST than 79266 and all other recombination types (Figure 3b). These results indicated that *qAHPS07* was located in the interval between *S7‐111* and *S7‐126*, with a physical distance of approximately 53.25 kb on chromosome A07 (Figure 3a).

To further verify and narrow down the genomic interval for *qAHPS07*, F_2:3_ progeny was created from individuals of the F_2_ recombinants D‐L (F_2:3_‐D to F_2:3_‐L) for further testing. Two new markers, *S7‐113* and *S7‐120*, were introduced for the interval between *S7‐111* and *S7‐126*. The results showed that F_2:3_‐D, E, I, J, K and L showed no significant difference in the four traits among the three genotypes, whereas significant differences in all four traits were observed between the three genotypes for F_2:3_‐F and F_2:3_‐J (Figures 3c,d and S7a,b). Based on the recombination breakpoints in F_2:3_‐E and F_2:3_‐L, the *qAHPS07* locus was narrowed down to a 36.46 kb region defined by the *S7‐113* and *S7‐126* interval on chromosome A07 (*Arahy*.07: 424670 bp ~ 461129 bp) (Figure 3c,d).

### Identification and sequence alignment of the candidate gene

**Figure 4:**
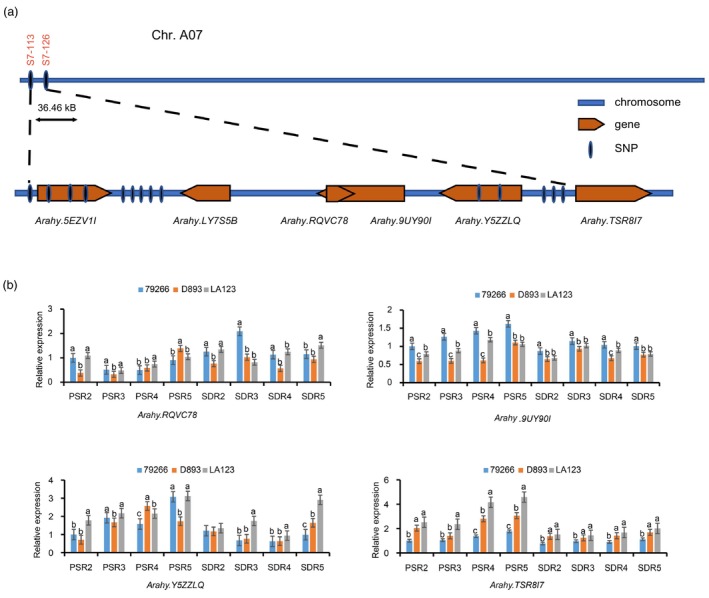
Candidate interval sequence analysis and gene expression analysis. (a) SNP locus and annotated genes within the QTL region. (b) The relative expression levels of *Arahy.9UY90I*, *Arahy.RQVC78*, *Arahy.Y5ZZLQ* and *Arahy.TSR8I7* in pod shell and seed of 79266, D893 and LA123 at four growth developmental stages. PSR2‐PSR5 represent R2 to R5 stages in the pod shell, SDR2‐SDR5 represent R2–R5 stages in the seed. Statistically significant differences are indicated by different lowercase letters (*P* < 0.05).

The 36.46 kb region contained the entire sequence of *Arahy.5EZV1I*, *Arahy.LY7S5B*, *Arahy.9UY90I*, *Arahy.RQVC78*, *Arahy.Y5ZZLQ* and the regulatory region of *Arahy.TSR8I7* (Figure 4a, Table S9). Based on the genome re‐sequencing data of 79266 and D893, we identified 14 SNPs in the 36.46 kb region. Of the 14 SNPs, 6 SNPs were intergenic, 3 SNPs were upstream and 5 SNPs were genic, including four intronic and one synonymous (Table S10). These results indicated that the significant difference of pod size between 79266 and D893 may be potentially due to differences in gene expression patterns.

Therefore, the expression patterns of the six candidate genes were tested in the pod shells and seeds of 79266, D893 and LA123, at the four developmental stages, R2‐R5. The results showed that *Arahy.5EZV1I* and *Arahy.LY7S5B* were not detected in 79266, D893 and LA123, and the expression of *Arahy.RQVC782* and *Arahy.Y5ZZLQ* in the shell and seed of the 79266, D893 and LA123 was not related to pod size. Interestingly, *Arahy.9UY90I* showed higher transcript levels in the shell and seed of 79266 than in D893 and LA123, and *Arahy.TSR8I7* showed a lower transcript level in the shell and seed of 79266 than in D893 and LA123 at all four growth stages (Figure 4b). In addition, no SNP loci were detected in the full‐length and regulatory region of the *Arahy.9UY90I* gene. However, three SNPs were detected in the promoter region of *Arahy.TSR8I7* (Figure 4a). Therefore, *Arahy.TSR8I7*, a RuvB‐like2 protein coding gene (Figure S8a), was identified as the potential candidate for *qAHPS07* and named as *AhRUVBL2*.

The CDS of *AhRUVBL2* were cloned from 79266 and D893, and there was not any difference at the nucleotide level (Figure S8b). Sequence alignment represented that the amino acid sequence of RUVBL2 in cultivated peanut share 100% similarity with that of *A*. *duranensis*, and showed similarity of more than 80% with RUVBL2s from other crops, human and danio‐rerio (Figure S9). Especially the Walker A, Walker B, Sensor 1, Sensor 2 and Trans‐Arginine fingers, which constitute the nucleotide binding motifs in the TIP49 domain (Silva *et al*., 2018), were highly conserved among all species(Figure S9). Subcellular localization of *AhRUVBL2* in *Arabidopsis* protoplast showed that it was located in the nucleus (Figure S8c).

Genome re‐sequencing results showed that there were 3 SNPs, *SNP_460907* *(G/A)*, *SNP_461023* *(A/T)* and *SNP_461129* *(C/T)*, in the promoter of *AhRUVBL2*, which were located at −437, −544 and −660 bp upstream of 5′UTR. The (A/T) variation of *SNP_461023* leads to a transition from ‘TAAA’ in 79266 to ‘TATA’ in D893 (Figure S10a). To investigate whether the new TATA‐box would create generate new transcription start site (TSS), the 5′RACE (rapid amplification of cDNA ends) was performed to amplify the 5′‐end of cDNA of 79266 and D893. Sequence analysis showed that the TSS of the *AhRUVBL2* gene in the two varieties were identical (Figure S10b,c).

### Function validation of the *AhRUVBL2* gene

**Figure 5:**
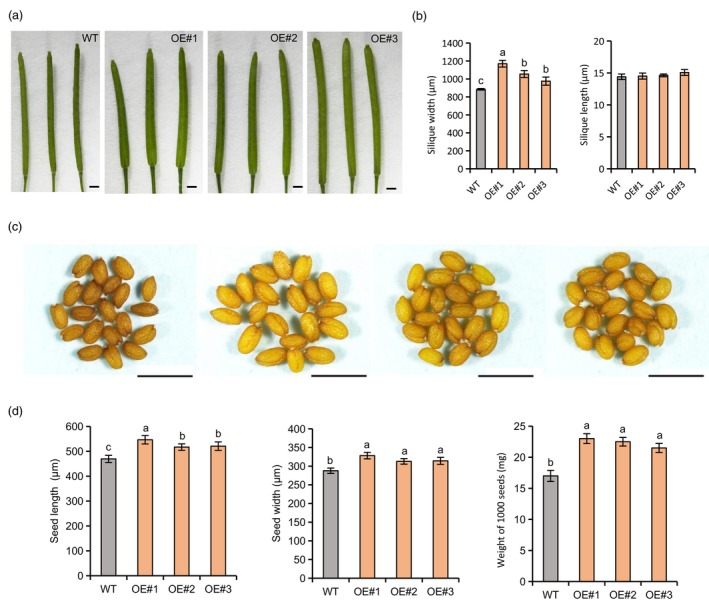
Phenotypic analysis of siliques and seeds in *AhRUVBL2* overexpression *Arabidopsis*. (a) Siliques from overexpression lines (OE#1 to OE#3) and wild type (WT). (b) Statistical data of silique width and silique length of WT and OE lines. (c) Seeds of OE lines and WT. (d) Statistical data of seed width, seed length and weight of 1000 seeds. Statistically significant differences are indicated by different lowercase letters (*P* < 0.05). Bar = 1 mm.

The CDS of *AhRUVBL2*, driven by CaMV 35S promoter, was introduced into *Arabidopsis* (*Col‐0*). Expression analysis showed that *AhRUVBL2* was expressed in transgenic plants, but not detected in wide type (WT) (Figure S11a). Compared with WT, the overexpression lines showed larger rosette diameter and higher plants (Figure S11b,c). The width of the transgenic silique was significantly larger than that of WT (Figure 5a,b). Maturation seeds of overexpression lines were significantly larger than WT in seed length, seed width and 1000‐seed weight (Figure 5c,d).

**Figure 6:**
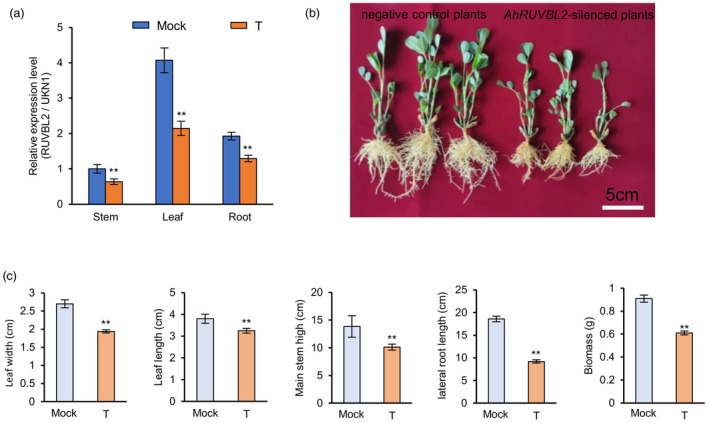
Phenotype analysis of *AhRUVBL2*‐silenced lines. (a) Relative expression levels of *AhRUVBL2* in three tissues from negative control (Mock) and *AhRUVBL2*‐silenced D893 (T) 14 days after infection. (b) Plants of negative control and *AhRUVBL2*‐silenced seedlings 14 days after infection. The left three are negative control plants, while the 3 on the right are *AhRUVBL2*‐silenced plants. (c) Statistical data of leaf width, leaf length, main stem high, lateral root length and biomass in negative controls (Mock) and *AhRUVBL2*‐silenced (T) seedlings 14 days after infection. Significant differences are indicated by * (*P* < 0.05), **(*P* < 0.01) (Student's *t‐*test).

Our research group has established a mature and efficient peanut whole plant VIGS system and verified its high efficiency in peanut gene silencing (Luo *et al*., 2021). Therefore, this system was used to explore the function of *AhRUVBL2* in peanut. To assay the validity and efficiency of this method, newly grown young leaves, stems and lateral roots were harvested to detect *AhRUVBL2* expression levels at 14‐days post‐inoculation. qRT‐PCR indicated that the expression of *AhRUVBL2* in silenced plants was significantly decreased compared with negative controls in leaves, lateral roots and stems (Figure 6a). Phenotypic analysis showed that silenced plants grew relatively slowly (Figure 6b), and the plant biomass, main stem height, leaf length, leaf width and lateral root length were significantly lower than those of the negative controls (Figure 6c).

### Genetic variations in the promoter of *AhRUVBL2* affect peanut pod size‐related traits

Based on the genome re‐sequencing data of the 119 cultivated peanut accessions, we identified three SNPs in promoter region of *AhRUVBL2* (*SNP_460907(G/A)*, *SNP_461023(A/T)* and *SNP_461129(C/T)*). The genome‐wide association study (GWAS) performing using a general linear model showed that these three SNPs were significantly associated with SPW (−log_10_ *P* = 5.28, 5.59, 5.49; *R* ^2^ = 0.10, 0.12, 0.12; Figure S12). Three haplotypes of *AhRUVBL2*, namely *Hap_GAC*, *Hap_ ATT* and *Hap_GAT*, were characterized in 119 peanut accessions by the differential contents of three SNPs. Among them, 78 lines carrying *Hap_GAC*, and 38 lines carrying *Hap_ATT*, which were consistent with 79266 and D893, respectively. The remaining 3 lines carried *Hap_GAC* and were consistent with *A*. *duranensis* (Table S11). These results indicated that the genomic loci of the three SNPs in cultivated peanut were closely linked and tended to be co‐inherited.

**Figure 7:**
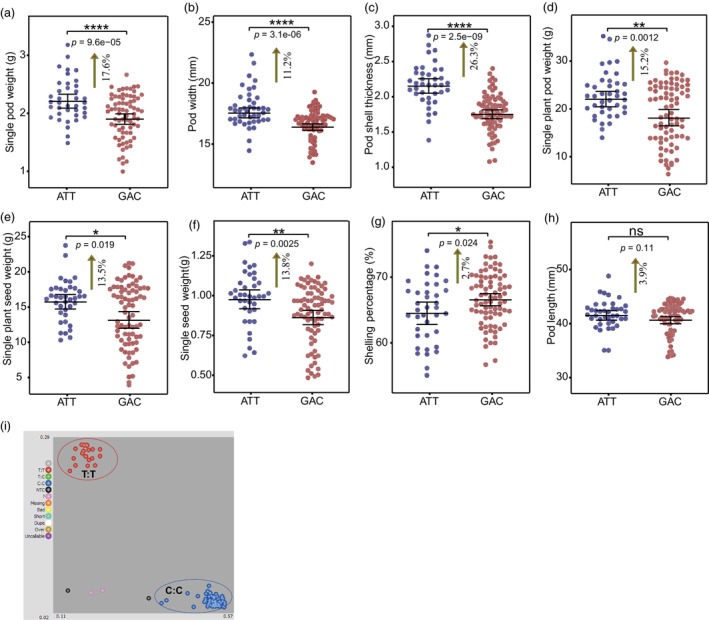
Haplotype analysis of *AhRUVBL2* gene based on the SNPs in the promoter region. Association analysis of two haplotypes (*Hap_ATT* and *Hap_GAC*) with single pod weight (a), pod width (b), pod shell thickness (c), single plant pod weight (d), single plant seed weight (e), single seed weight (f), shelling percentage (g) and pod length (h) among 119 peanut accessions. (i) Genotyping results of *SNP_461129* by KASP in 119 cultivated peanut accessions. The scatter plot with axes x and y represents allelic discrimination of this site accessions. The red and blue dots represent the D983 homozygous and 79266 homozygous, respectively. ATT represents *Hap_ATT* Haplotype; GAC represents *Hap_GAC* Haplotype. Significant differences are indicated by *(*P* < 0.05), **(*P* < 0.01), ****(*P* < 0.0001), ‘ns’ stand for not significant. (Student's *t‐*test).

Phenotype analysis showed that accessions carrying *Hap_ATT* represented significant higher (11.2%–26.3%) SPW, PW, PST, single plant pod weight, single plant seed weight and single seed weight compared with those carrying *Hap_GAC* (Figure 7a‐f). However, shelling percentage of accessions carrying *Hap_ATT* was 2.7% lower than others (Figure 7g), while no significant (*P* = 0.11) difference was detected in PL between haplotypes (Figure 7h). These results indicate that *Hap_ATT* is a dominant haplotype associated with pod size. In addition, a KASP marker ‘*S7‐126*’ was developed from *SNP_461129* to identify *Hap_GAC* and *Hap_ATT* haplotypes, which provides application basis for marker assisted selection (MAS) in peanut breeding (Figure 7i).

## Discussion

### The chromosome A07 terminal genome region of peanut is a strongly correlated region controlling pod development and size

Several QTLs for pod or seed‐related traits have been mapped on chromosome A07 using RIL or CSSL populations. Two major QTLs and a QTL cluster are located within a 1.48 Mb region between 0.06 Mb to 1.54 Mb (Gangurde *et al*., 2020; Li *et al*., 2021; Luo *et al*., 2018), and another two major QTLs and a QTL cluster were located within a 1.27 Mb region between 0.87 Mb to 2.14 Mb (Alyr *et al*., 2020; Chen *et al*., 2017; Zhuang *et al*., 2019). QTLs for various traits with positive or negative correlations with each other tend to co‐localize within the same or adjacent genomic regions, which suggests that pleiotropism gene or gene linkage exist in that chromosome region (Wang *et al*., 2020a). The phenomenon of linked distribution of genes related to fruit or seed development is widespread in crops. For example, *GS5*, *GW5* and *chalk5* related to seed development are closely linked on chromosome 5 of rice, and their minimum distance is less than 110 kb (Li *et al*., 2011, 2014; Weng *et al*., 2008). These above studies indicated that the terminal region of chromosome A07 does have major and pleiotropic gene or closely linked gene clusters related to pod development. However, the cloning of key genes and regulatory mechanisms in chromosome A07 are rarely reported.

In the present study, we fine‐mapped a major QTL, *qAHPS07*, within a 0.04 Mb region (*Arahy*.07: 0.42–0.46 Mb) using F_2_, RIL and secondary F_2_ populations, which explained 38.6% of the phenotypic variation for SPW, 23.35% for PL, 37.48% for PW and 25.94% for PST (Figures 2 and 3). Compared with previous studies, the accuracy of QTL interval was greatly improved. In addition, we found an interesting phenomenon that there was no significant correlation between the PST and PL in the F_2_ population, but their significant correlation was observed in the RIL population (Tables S3 and S4). Corresponding to this, a minor QTL for PL was detected in interval *S7‐19* to *S7‐22* in F_2_ population (Table S7). This discrepancy might be explained by the control of PST and PL by a group of closely linked genes in chromosome A07, which would be less frequently dissociated by chromosomal segregation, thus requiring the production of a large number of progenies to be observed. Therefore, the terminal region of chromosome A07 in peanut should continue to be paid more attentions in peanut breeding programs.

### *AhRUVBL2* is a positive regulator of peanut pod size by affecting cell division of pod shell

Previous studies have identified many QTLs for peanut pod size; however, because the number of candidate genes within the loci are more than dozens, gene cloning and function analysis are rarely studied. In this work, we found 6 candidate genes (Figure 4a), and confirm that *AhRUVBL2* is the key regulatory gene of pod size. Studies in animals have shown that *RUVBL2* plays a key role in cell cycle progression, RNA polymerase II directed transcription and DNA damage response, and that the expression level of *RUVBL2* is the dominant factor regulating the cell cycle or cell proliferation (Jin *et al*., 2005; Kanemaki *et al*., 1999). The proliferation rate of cancer cells was observed to slow down significantly with a decrease in expression of *RUVBL2* (Jin *et al*., 2005; Kanemaki *et al*., 1999; Rousseau *et al*., 2007; Silva *et al*., 2018). Usually, highly conserved genes have similar functions. We found that the amino acid sequence of RUVBL2 proteins in various plants and animal species are highly similar (Figure S9), which suggests that function of plant *RUVBL2* may be similar to that in animals, that is regulating cell cycle and/or cell proliferation.

In many crops, the number and size of hull cells affect the shape, size and weight of fruits or seeds (Hu *et al*., 2018; Huang *et al*., 2017; Liu *et al*., 2015; Xu *et al*., 2018). In rice, several genes have been identified affecting grain size or weight by regulating spikelet hull cell division (Song *et al*., 2007; Sun *et al*., 2018; Xu *et al*., 2018) or cell expansion (Hu *et al*., 2018; Huang *et al*., 2017; Xu *et al*., 2018). In rapeseed, *ARF18* regulates silique length and grain weight by regulating cell expansion in the silique wall (Liu *et al*., 2015). In this study, we found that the number of pod shell cells was the main reason for the difference in pod size between 79266 and D893. From the R4 stage, cotyledon cells of D893 expanded more rapidly than those of 79266, which lead to larger cells (Figure 1). These observations suggest that the larger pod shell of D893 provided sufficient space for the development of the seeds. Moreover, the expression of *AhRUVBL2* in pod shell is significantly higher than that in seed (Figure 4b). Therefore, *AhRUVBL2* may primarily regulate the proliferation of pod shell cells. Its high expression in pod shells of D893 enhances cell proliferation ability, which lead to increased cell numbers, ultimately making the pod larger than 79266.

Interestingly, one of the six candidates identified in this work, *Arahy.5EZV1I* is heavily involved in the control of peanut seed size in peanut (Zhuang *et al*., 2019). However, the expression of *Arahy.5EZV1I* was not detected in 79266 and D893, which probably reflects differences in genetic background between accessions, and indicates pod size is controlled by complex mechanisms that are rarely understood.

### Application of *AhRUVBL2* in peanut breeding

In the process of plant evolution, natural mutation and artificial selection often occur. Natural genetic variation makes its genetic diversity richer and artificial selection can change gene frequency directionally (Chen *et al*., 2022). In the present study, three SNPs, *SNP_460907*, *SNP_461023* and *SNP_461129*, were found in the *AhRUVBL2* promoter region (Figure S10a). These three SNPs provided the basis for the classification of the 119 peanut accessions into three haplotypes, including 78 lines carrying *Hap_GAC*, 38 lines carrying *Hap_ATT* and 3 lines carrying *Hap_GAT* (Table S11). The *Hap_GAT* was consistent with that of wild peanut (*A. duranensis*), suggesting that *Hap_GAC* and *Hap_ATT* were generated by natural mutations during the domestication of cultivated peanut. The presence of only three lines carrying *Hap_GAT* in the 119 peanut accessions suggests that there was strong artificial selection applied during the domestication of cultivated peanut, resulting in a decrease in the *Hap_GAT* frequency.

More importantly, haplotype analysis among *Hap_GAC* and *Hap_ ATT* showed that, accessions carrying *Hap_ATT* represented significant higher SPW, PW, PST, single plant pod weight, single plant seed weight and single seed weight (Figure 7a–f), which revealed that the function of *AhRUVBL2* is universally applicable among accessions. Although the SP of accessions carrying *Hap_ ATT* was slightly lower than that of *Hap_GAC*, there were still some accessions with higher SP that could be used for breeding selection (Figure 7g). However, the frequency of *Hap_ATT* in currently cultivated peanut remains low, and increasing the frequency of *Hap_ATT* would play an important role in peanut breeding. By using MAS, favourable haplotypes can be selected to achieve desirable pod features in peanut (Varshney *et al*., 2014a). In this study, a KASP marker developed from *SNP_461129* (Figure 7i) will provide an application basis for future genetic improvement of peanut that needs to be done from the pod size aspect.

## Conclusion

In this study, a major QTL related to pod size, *qAHPS07*, was fine mapped to a 36.46 kb interval on chromosome A07. The evidences of forward genetics and reverse genetics all strongly indicated that *AhRUVBL2* (*Arahy.TSR8I7*) was the most likely candidate gene for *qAHPS07*. *AhRUVBL2* may regulate the proliferation of pod shell cells, which ultimately affects the pod size. Three haplotypes of *AhRUVBL2* were characterized in 119 cultivated peanut accessions by three SNPs in the promoter region. *Hap_ATT* is a dominant haplotype associated with pod size. In addition, a tightly linked molecular marker of *AhRUVBL2*, which could be used for genetic improvement of peanut pod size‐related traits.

## Experimental procedures

### Plant materials and field trials

The F_2_ and RIL populations used in this study consisted of 1020 individuals and 151 lines, respectively, were derived from a cross between the varieties 79266 (as the female, small pod) and D893 (as the male, big pod; Li *et al*., 2017). The RILs together with the parent lines were planted in Taian, China in 2011 (F_6_ generation; E1), 2012 (F_7_ generation; E2), 2013 (F_8_ generation; E3), 2014 (F_9_ generation; E4) and 2015 (F_10_ generation; E5), as well as in Jining, China in 2013 and 2014 (F_8_ generation; E6 and F_9_ generation; E7, respectively). The F_2_ population was planted in Taian, China in 2017.

LA123 is an inbred line from a RIL population (F_13_ generation) that had super‐larger pods, which exhibited a D893 genotype within the candidate interval of *qAHPS07*. The SSR genetic map constructed by RIL population showed that the other genetic background of LA123 was similar to 79266 (Li *et al*., 2017). The secondary F_2_ population consisting of 1039 plants was developed from the backcross of 79266 with LA123. The secondary F_2_ population were planted in Taian, China in 2019. The progeny of recombinant plants (F_2:3_) from the secondary F_2_ population were planted in Taian, China in 2020.

The 119 cultivated peanut accessions containing 60 lines of var. *hypogaea* and 59 lines of irregular types were used for gene effect verification. These accessions included main cultivars, important breeding parents and advanced breeding lines (Zhang *et al*., 2018). The 119 peanut accessions were planted in Taian, China in 2019.

### Trait measurements and pod sample collection

SPW, PL, PW and PST of mature pods were measured after harvest of the RIL, F_2_, secondary F_2_ and secondary F_2:3_ populations. The traits were determined from 5 representative pods selected from each of the 151 RILs, F_2_, secondary F_2_ and secondary F_2:3_ populations. In addition to the above traits, seed weight, single plant pod weight, single plant seed weight and SP were also investigated in the 119 peanut accessions according to the published standard procedures (Luo *et al*., 2017). BLUPs for pod size‐related traits of the RIL population in the seven environments were calculated using the package lme4 (Bates *et al*., 2015) in R. Basic statistical analysis, phenotypic correlation coefficient and analysis of variance were calculated using SPSS version 20.0 (IBM SPSS, Chicago, IL).

Only after compartment pods were taken and the shells and seeds were separated. Pod samples at the R2 stage were collected 30 days after flowering, and were collected weekly until the R5 stage. The development stages were estimated according to previous reports (Boote, 1982; Zhang *et al*., 2022b). 40 representative pods from 15 individual plants were collected at each sampling time point and divided into 4 replicates, with 10 pods per replicate.

### Microscopic analyses

Peanut pod shells and seeds were collected at the R2‐R5 stage, and immediately fixed for 24 h at 4 °C in formalin‐acetic acid‐alcohol (FAA). Paraffin sections were prepared according to previous report (Zhang *et al*., 2016). Pod shells were stained with safranin O/fast green (Solarbio, Beijing Solarbio Technology Co., Ltd.) and seeds were stained with fast green. All samples were scanned using a NIKON ECLIPSE E100 microscope (Nikon Instruments, Japan). Image J software (Schneider *et al*., 2012) was used to measure the cell area of pod shells and seeds. The total number of cell layers in the exocarp and mesocarp of the pod shell was calculated as N = T/W, where T is the thickness of the exocarp and mesocarp of the pod shell, W is the mean width of cells in the exocarp and mesocarp of the pod shell. Total number of cells in cotyledons transection was calculated as n = π(d/2)^2^/a, where d is the width of the seed and a is mean cell area in the seed cotyledon.

### Whole genome re‐sequencing and BSA‐seq analysis

For developing the extreme bulks, leaves derived from 30 plants with big or small pods were selected from the F_2_ population based on their phenotypic values of SPW. Genomic DNA of the bulks and two parents were extracted using Plant Genomic DNA Extraction Kit (TIANGEN, Beijing, China) according to the manufacturer's instructions. Big pod and small pod bulks were constructed by mixing an equal amount of DNA from 30 big pods and 30 small pods, respectively. Pair‐end sequencing libraries (read length 150 bp) with insert sizes of approximately 250–350 bp were prepared for sequencing with an Illumina HiSeq2500 system. After sequencing, clean reads and SNPs were obtained according to Zhang *et al*. (2022a). SNPs homozygous and polymorphic between the parents were selected for BSA‐seq. The SNP index and Δ(SNP‐index) for each SNP position for both bulks were calculated using the statistical method described by Takagi *et al*. (2013), where SNPindex (s/b) = Ms/b/(Ms/b + Ps/b), ΔSNP‐index = SNPindex(s)‐ SNPindex (b), where s and b represent the small pod and big pod bulks, respectively. M and P are the numbers of reads harbouring a SNP that was compared to the reference genome of 79266 and D893, respectively. The genomic regions, whose ΔSNP‐index were significantly higher than the threshold at the 99% level of significance were considered as candidates controlling pod size (Luo *et al*., 2019b).

### KASP marker development, genetic map construction and QTL mapping

In order to confirm the predicted candidate genomic regions and to fine map pod size‐related QTLs, KASP markers (He *et al*., 2014a) for 174 SNPs between the 79266 and D893 cultivars were developed in the candidate region by BSA‐seq. For each KASP marker, two allele‐specific forward primers and one common reverse primer were designed based on 100 bp upstream and downstream sequences of genic SNPs in LGC Genomic Ltd. Hoddesdon, UK (Table S6).

The genetic linkage map was constructed using JoinMap4.0 software (JoinMap^®^ 4.0: Software for the calculation of genetic linkage maps in experimental populations, https://www.kyazma.nl/index.php/JoinMap/). The linkage map was present using MapChart 2.3 software (Voorrips, 2002). ICIMmapping 4.0 software was used for inclusive composite interval mapping (ICIM) to identify and analyse QTLs (Meng *et al*., 2015). Using this method, a LOD ≥3.0 was considered indicative of a QTL.

### Prediction of candidate genes

The reference genome of *Arachis hypogaea* cv. tifrunner (Bertioli *et al*., 2019; version 1 available at https://peanutbase.org) and SnpEff 4.2 software (Cingolani *et al*., 2012) were used to locate genomic variants and predict their effects on known genes at loci containing QTLs. The functions of the candidate genes were further annotated with KOG (https://www.ncbi.nlm.nih.gov/research/cog) GO (https://www.geneontology.org) and KEGG (http://www.genome.jp).

### Total RNA extraction and quantitative real‐time PCR (qRT‐PCR)

Total RNA for all the samples was extracted using the EasyPure Plant RNA Kit (TranGen Biotech, Beijing, China). Reverse transcription was performed using the Prime Script™ RT Reagent Kit (TaKaRa, Inc., Dalian, China). The qRT‐PCR was performed using an SYBR^®^Premix EX Taq™Kit (TaKaRa, Inc., Dalian, China) on Step One Plus Real‐Time PCR (ABI, USA), with the following protocol: 95 °C for 300 s, followed by 40 cycles at 95 °C for 20 s, 60 °C for 20 s, 72.0 °C for 20 s. The relative expression levels of each gene among different samples were calculated using the2^−▵▵Ct^ method (Livak and Schmittgen, 2001) and normalized by the internal reference *UKN1* (EG028875, AT3G13410) gene (Chi *et al*., 2012). Three biological replicates of each sample type were collected and three technical replicates were performed for each biological replicate.

### 5′ race

The transcription start site of *AhRUVBL2* gene was identified by 5′RACE (rapid amplification of cDNA ends) using a 5′RACE kit (5′RACE System for Rapid Amplification of cDNA Ends, Version 2.0, Invitrogen). Total RNA was obtained from the pod shells of 79266 and D893 at the R5 developmental stage, respectively. Three gene‐specific Primers, *GSP1*, *GSP2* and *GSP3* (Table S12) were design by Premier Primer 5.0 software (Premier Biosoft International, CA). First‐strand cDNA of the *AhRUVBL2* gene was synthesized from total RNA using SUPERSCRIPT II RT enzyme and *GSP1*. *GSP2* and *GSP3* were used for the primary PCR and nested PCR, and these two PCR were performed according to a previous study (Tang *et al*., 2021). The nested PCR products were collected for sequencing.

### Subcellular localization analysis of AhRUVBL2 in *Arabidopsis* protoplasts

The coding sequence fragments of *AhRUVBL2* (Table S12) without the stop codon was inserted into 35S::GFP vector p16318 by using restriction endonuclease Hind III and Xba I site to construct *35S*:: *AhRUVBL2*‐GFP. The empty vector p16318 (35S::GFP) were used as control. The preparation of *Arabidopsis* protoplasts was according to a previous study (Yoo *et al*., 2007). The transformed protoplasts were cultured in 25 °C under darkness for 14–20 h and GFP was observed by confocal laser scanning microscopy at 488 nm (LSM800, Zeiss, Germany).

### Transformation of *Arabidopsis* for overexpression of *AhRUVBL2*

The coding sequence of *AhRUVBL2* (Table S12) was inserted into the pCAMBIA1300‐35S vector to generate the construct pCAMBIA1300‐35S‐*AhRUVBL2*. The resultant vectors were transformed into *Agrobacterium tumefaciens* GV3101 and infect *Arabidopsis* (Col‐0) using the floral dip method (Clough and Bent, 1998). T_3_ generation homozygous transgenic *Arabidopsis* lines were used for further analysis. For siliques and seeds size analysis, 20 randomly samples were harvest from WT and transgenic lines, respectively. Silique length and width were measured by vernier callipers. Seeds were photographed under a microscope, and Image J software was used to determine the seed length and width. The 1000 seed weights of WT and transgenic lines were determined from randomly selected seeds using an electronic balance (±0.0001 g).

### Whole plant virus‐induced gene silencing of *AhRUVBL2*

The effects of *AhRUVBL2* gene silencing were investigated using the VIGS system with tobacco rattle virus (TRV) (Liu *et al*., 2002), using whole plant VIGS system described for peanut (Luo *et al*., 2021). Part of the CDS of *AhRUVBL2* (303 bp) (Table S12) was PCR‐amplified from peanut cDNA and cloned into the pTRV2 vector to generate the plasmids pTRV2‐*AhRUVBL2*. The pTRV1, pTRV2, and pTRV2‐*AhRUVBL2* vectors were transformed into *A. tumefaciens* GV3101 competent cells by the freeze–thaw method. The bacterial cultures harbouring pTRV1 and/or pTRV2/pTRV2 derivatives were mixed in a 1:1 (v/v) ratio and infiltrated into cotyledons of 5‐day old peanut seedlings using vacuum infiltration. The infiltrated seedlings were maintained under dark for 16 h and then grown in a culture room with a 16/8 h light/dark cycle at 25 °C. Two weeks after infection, plants carrying pTRV1 + pTRV2‐*AhRUVBL2* or pTRV1 + pTRV2 (negative control) were used for further analysis. At least 100 negative control plants and silenced plant seedlings each were vacuum infiltrated for every independent biological replicate.

## Conflict of interest

The authors declare that they have no conflict of interest.

## Author contributions

FZL and YSW conceived and designed the experiments. HY, LL, YYL, HDL, XRZ, KZ, SQZ, XLL and YJL performed the experiments. HY and LL analysed the data. HY drafted the manuscript. FZL and LL revised the manuscript. All authors discussed the results and commented on the article.

## Supporting information


**Figure S1.** Phenotypic distributions of single pod wight, pod length, pod width and pod shell thickness in the RIL population across seven environments (E1‐E7).

**Figure S2.** Phenotypic distributions of single pod wight, pod length, pod width and pod shell thickness in the F_2_ population.

**Figure S3.** The Δ(SNP‐index) plot obtained by subtraction of small bulk SNP‐index from big bulk SNP‐index.

**Figure S4.** SNP‐index plots for 20 pseudomolecules of big bulk.

**Figure S5.** SNP‐index plots for 20 pseudomolecules of small bulk.

**Figure S6.** Comparison of chromosome segments of LA123 with 79266 and D893. 1–20 represents the 20 chromosomes of the peanut genome.

**Figure S7.** The progeny test of four type recombinants.

**Figure S8.** CDS alignment and coding protein analysis of *AhRUVBL2* gene.

**Figure S9.** Sequence alignment analyses of RUVBL2 protein in plants and animals.

**Figure S10.** Identification of *AhRUVBL2* transcription start site by 5'RACE assay in 79266 and D893.

**Figure S11.** Phenotypic analysis of transgenic *Arabidopsis*.

**Figure S12.** GWAS of single pod weight in 119 cultivated peanut accessions.

**Table S1.** Descriptive statistical analysis of phenotypes of pod‐related traits in the RIL population.

**Table S2.** Descriptive statistical analysis of phenotypes of pod‐related traits in the F_2_ population.

**Table S3.** Correlation analysis of the pod size‐related traits in F_2_ population.

**Table S4.** Correlation analysis of the pod size‐related traits in RIL population by BLUP.

**Table S5.** The quality, sequencing depth and coverage of re‐sequencing data of parental lines and bulks.

**Table S6.** KASP markers used in this study.

**Table S7.** QTLs identified for pod size‐related traits in the F_2_ populations across seven environments.

**Table S8.** QTLs identified for pod size‐related traits in the RIL populations across seven environments.

**Table S9.** Information about the six candidate genes and primers were used in qRT‐PCR analysis.

**Table S10.** Summary of SNP_S_ of parental lines in the candidate interval.

**Table S11.** SNP_S_ information in promoter of *AhRUVBL2* in 119 cultivated peanut accessions.

**Table S12.** Genespecific primers for *AhRUVBL2*.
