# Supplementary material for: Fine mapping of qAHPS07 and functional studies of AhRUVBL2 controlling pod size in peanut (Arachis hypogaea L.)
Source: Plant Biotechnol J. 2023 May 31;21(9):1785–98. doi: 10.1111/pbi.14076 (PMC10440995; doi:10.1111/pbi.14076)
Supplement: Supplementary file 1 — Figure S1. Phenotypic distributions of single pod wight, pod length, pod width and pod shell thickness in the RIL population across seven environments (E1‐E7). [file PBI-21-1785-s007.pdf]

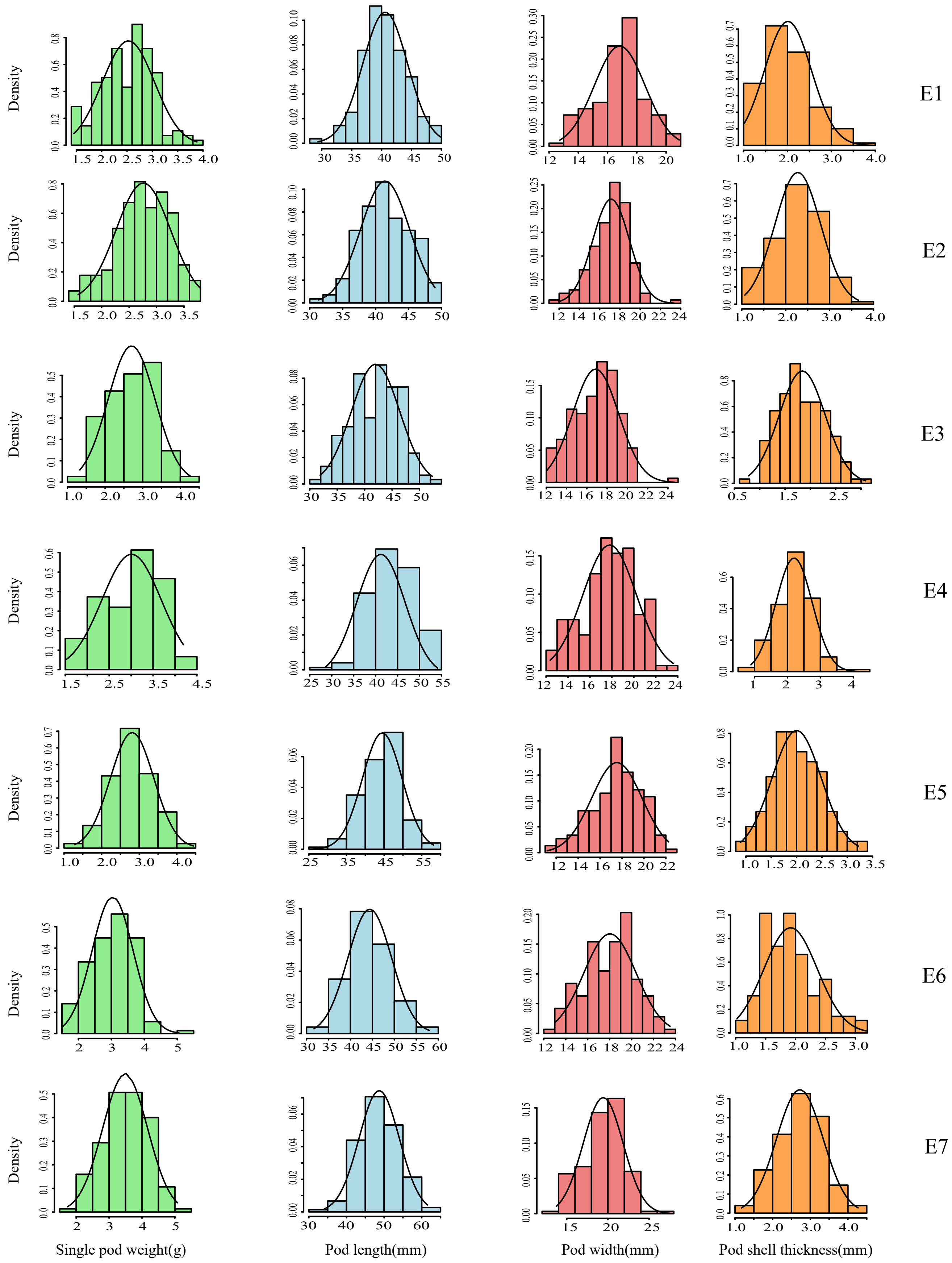

Figure S1 Phenotypic distributions of single pod wight, pod length, pod width and pod shell thickness in the RIL population across seven environments (E1-E7).
