# Supplementary material for: Fine mapping of qAHPS07 and functional studies of AhRUVBL2 controlling pod size in peanut (Arachis hypogaea L.)
Source: Plant Biotechnol J. 2023 May 31;21(9):1785–98. doi: 10.1111/pbi.14076 (PMC10440995; doi:10.1111/pbi.14076)
Supplement: Supplementary file 3 — Figure S3. The Δ(SNP‐index) plot obtained by subtraction of small bulk SNP‐index from big bulk SNP‐index. [file PBI-21-1785-s015.pdf]

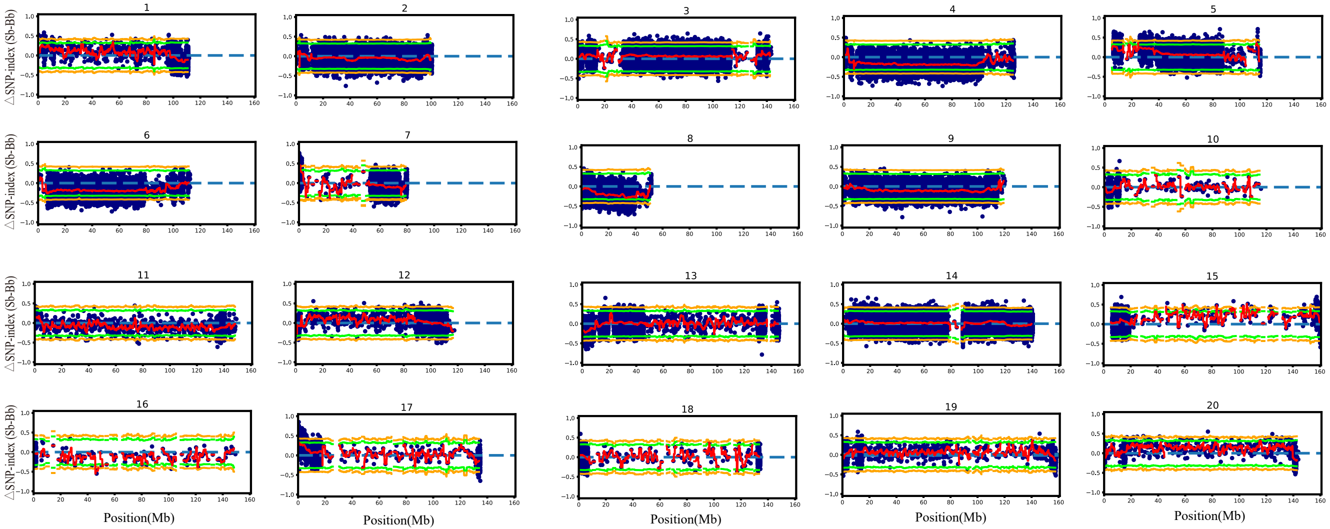

Figure S3 The Δ(SNP-index) plot obtained by subtraction of small bulk SNP-index from big bulk SNP-index.
