# Supplementary figures and images for: Fine mapping of qAHPS07 and functional studies of AhRUVBL2 controlling pod size in peanut (Arachis hypogaea L.)
Source: Plant Biotechnol J. 2023 May 31;21(9):1785–98. doi: 10.1111/pbi.14076 (PMC10440995; doi:10.1111/pbi.14076)

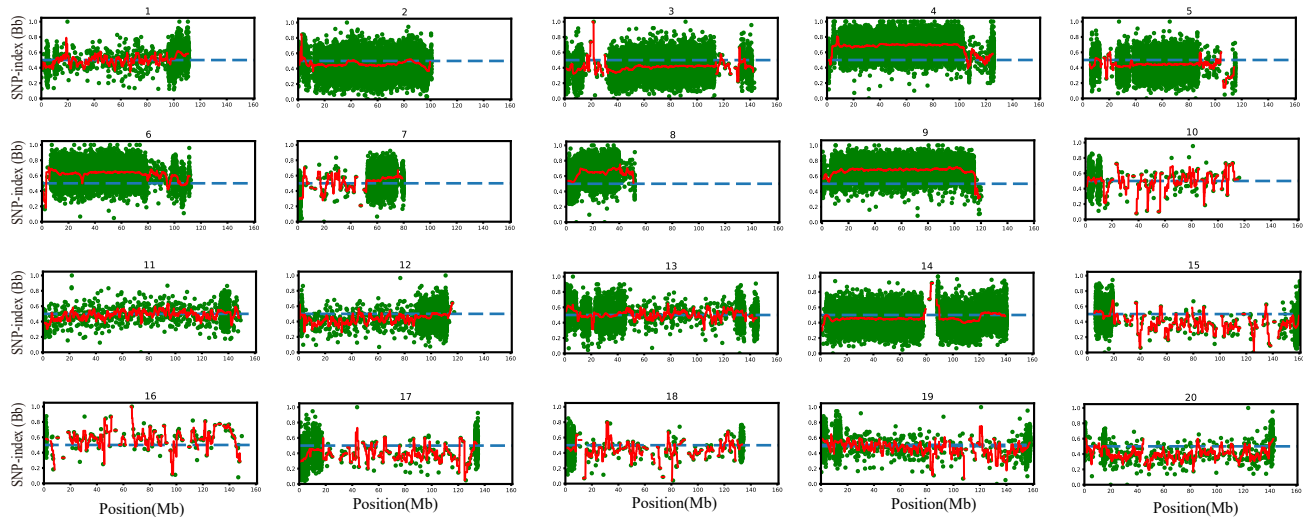

Figure S4 SNP-index plots for 20 pseudomolecules of big bulk.

Supplement: Supplementary file 4 — Figure S4. SNP‐index plots for 20 pseudomolecules of big bulk. [file PBI-21-1785-s011.pdf]

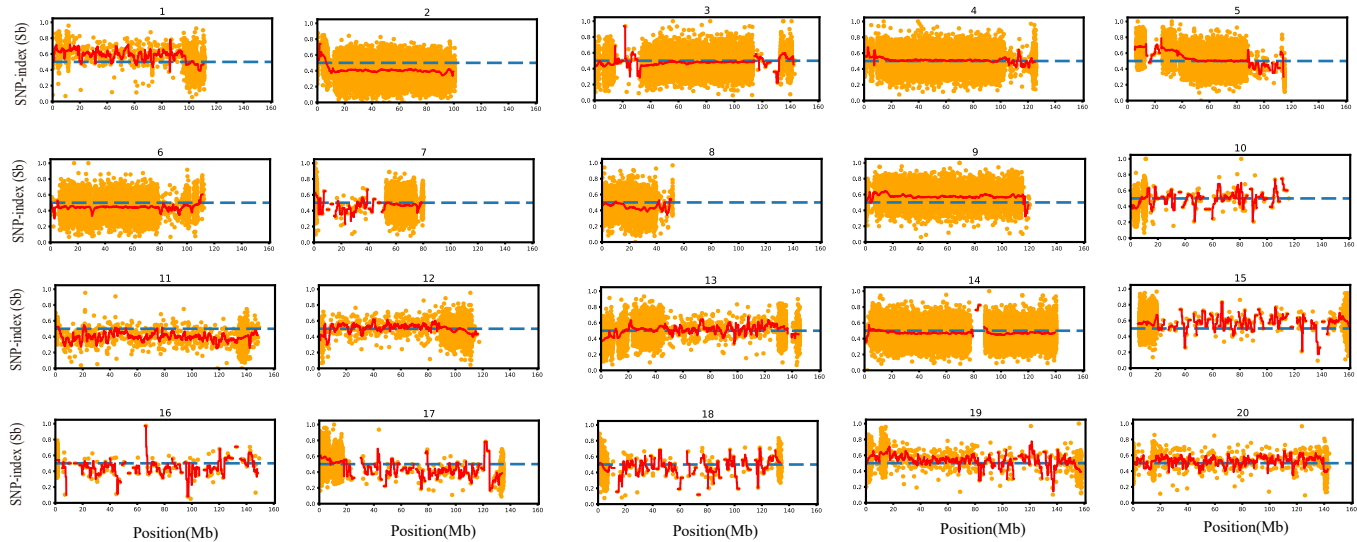

Figure S5 SNP-index plots for 20 pseudomolecules of small bulk.

Supplement: Supplementary file 5 — Figure S5. SNP‐index plots for 20 pseudomolecules of small bulk. [file PBI-21-1785-s024.pdf]
