# Supplementary material for: Fine mapping of qAHPS07 and functional studies of AhRUVBL2 controlling pod size in peanut (Arachis hypogaea L.)
Source: Plant Biotechnol J. 2023 May 31;21(9):1785–98. doi: 10.1111/pbi.14076 (PMC10440995; doi:10.1111/pbi.14076)
Supplement: Supplementary file 6 — Figure S6. Comparison of chromosome segments of LA123 with 79266 and D893. 1–20 represents the 20 chromosomes of the peanut genome. [file PBI-21-1785-s005.pdf]

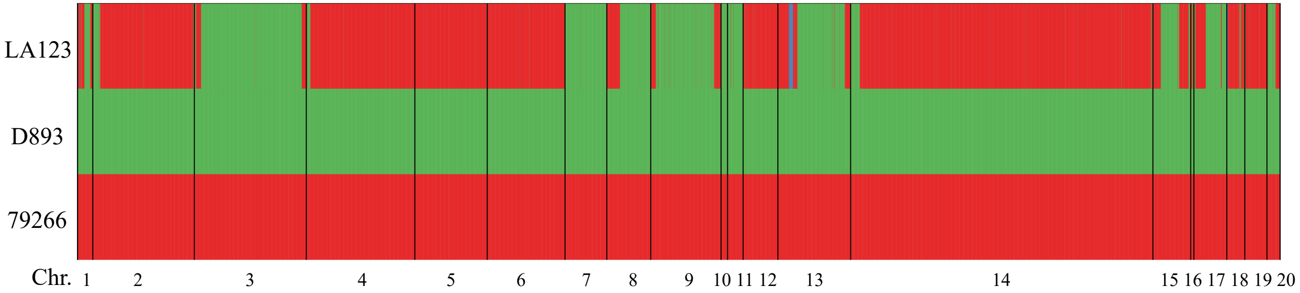

Figure S6 Comparison of chromosome segments of LA123 with 79266 and D893 . 1 – 20 represents the 20 chromosomes of the peanut genome.
