# Supplementary material for: Fine mapping of qAHPS07 and functional studies of AhRUVBL2 controlling pod size in peanut (Arachis hypogaea L.)
Source: Plant Biotechnol J. 2023 May 31;21(9):1785–98. doi: 10.1111/pbi.14076 (PMC10440995; doi:10.1111/pbi.14076)
Supplement: Supplementary file 7 — Figure S7. The progeny test of four type recombinants. [file PBI-21-1785-s019.pdf]

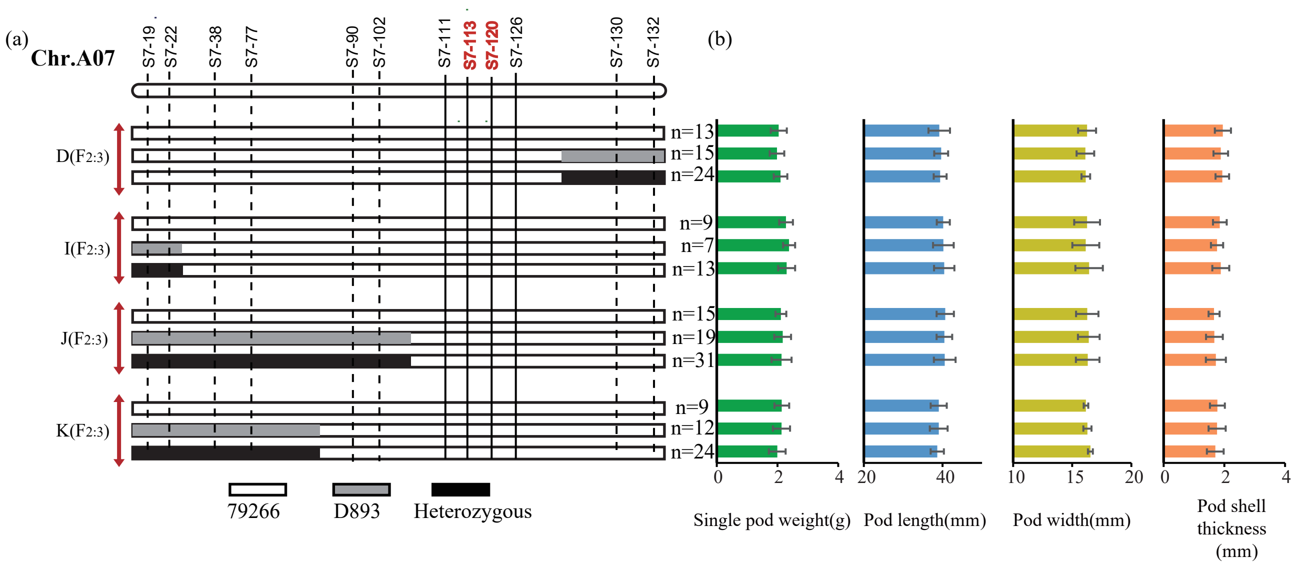

Figure S7 The progeny test of four type recombinants. (a) upside is the 12 markers used to screen secondary $F_{2:3}$ individual plants. n represent the numbers of individual plants of each type. White, gray and black bars represent 79266, D893 and heterozygous haplotypes, respectively. (b) The statistical data of SPW, PL, PW and PST of each type. Significant differences are indicated by * (P< 0.05), **(P < 0.01) (Student's t-test).
