# Supplementary material for: Fine mapping of qAHPS07 and functional studies of AhRUVBL2 controlling pod size in peanut (Arachis hypogaea L.)
Source: Plant Biotechnol J. 2023 May 31;21(9):1785–98. doi: 10.1111/pbi.14076 (PMC10440995; doi:10.1111/pbi.14076)
Supplement: Supplementary file 8 — Figure S8. CDS alignment and coding protein analysis of AhRUVBL2 gene. [file PBI-21-1785-s023.pdf]

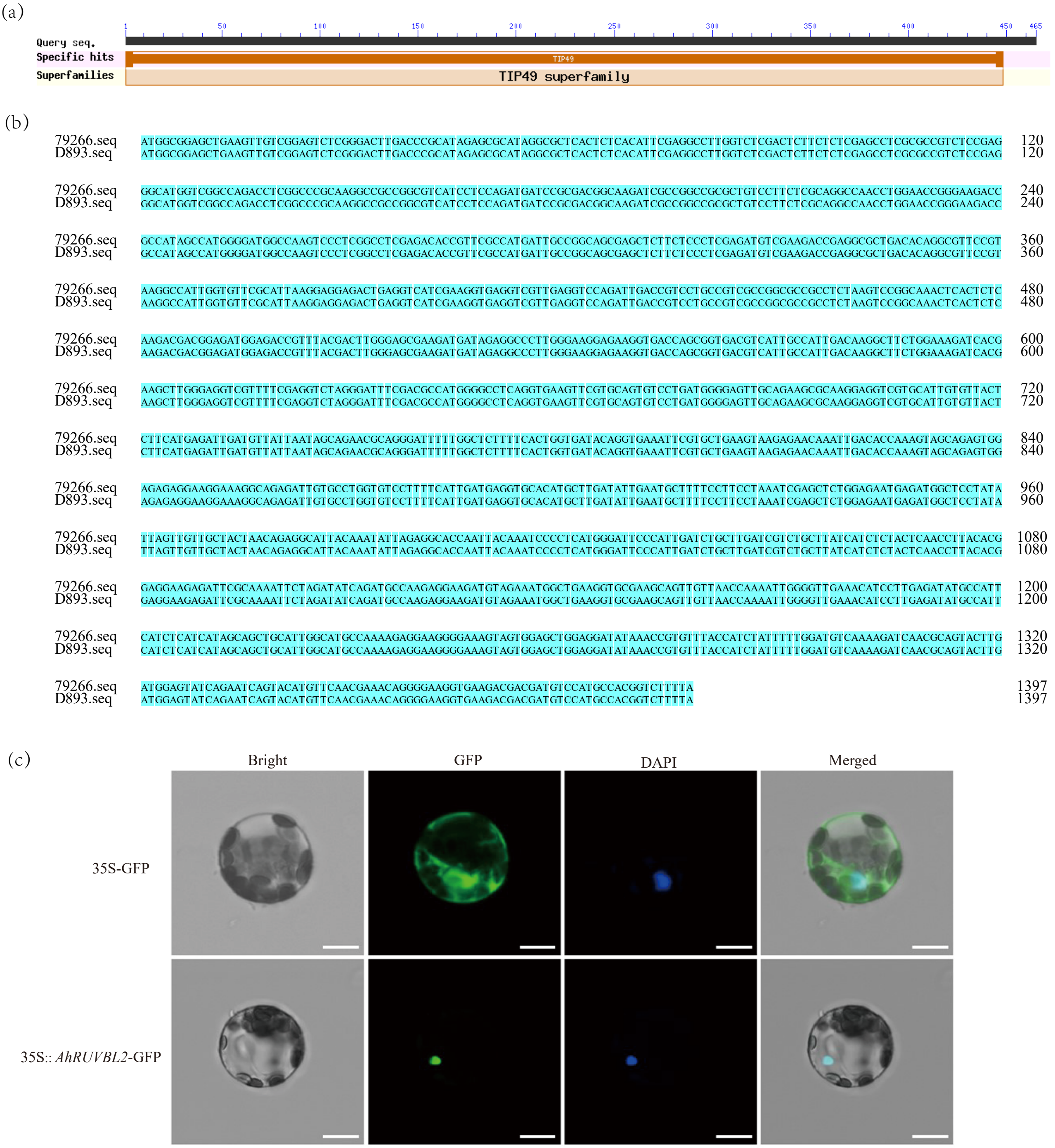

Figure S8 CDS alignment and coding protein analysis of *AhRUVBL2* gene.(a) Domain analysis of protein encoded by *AhRUVBL2*.(b) CDS alignment of *AhRUVBL2* gene in 79266 and D893.(c) Subcellular localization of *AhRUVBL2* in *Arabidopsis thaliana* protoplast, the vector control (35S::GFP) and fusion protein vector (35S::*AhRUVBL2*-GFP) were each introduced into *Arabidopsis thaliana* protoplasts. GFP was observed with laser scanning confocal microscope. Scale bar = 10 μm.
