# Supplementary material for: Fine mapping of qAHPS07 and functional studies of AhRUVBL2 controlling pod size in peanut (Arachis hypogaea L.)
Source: Plant Biotechnol J. 2023 May 31;21(9):1785–98. doi: 10.1111/pbi.14076 (PMC10440995; doi:10.1111/pbi.14076)
Supplement: Supplementary file 9 — Figure S9. Sequence alignment analyses of RUVBL2 protein in plants and animals. [file PBI-21-1785-s013.pdf]

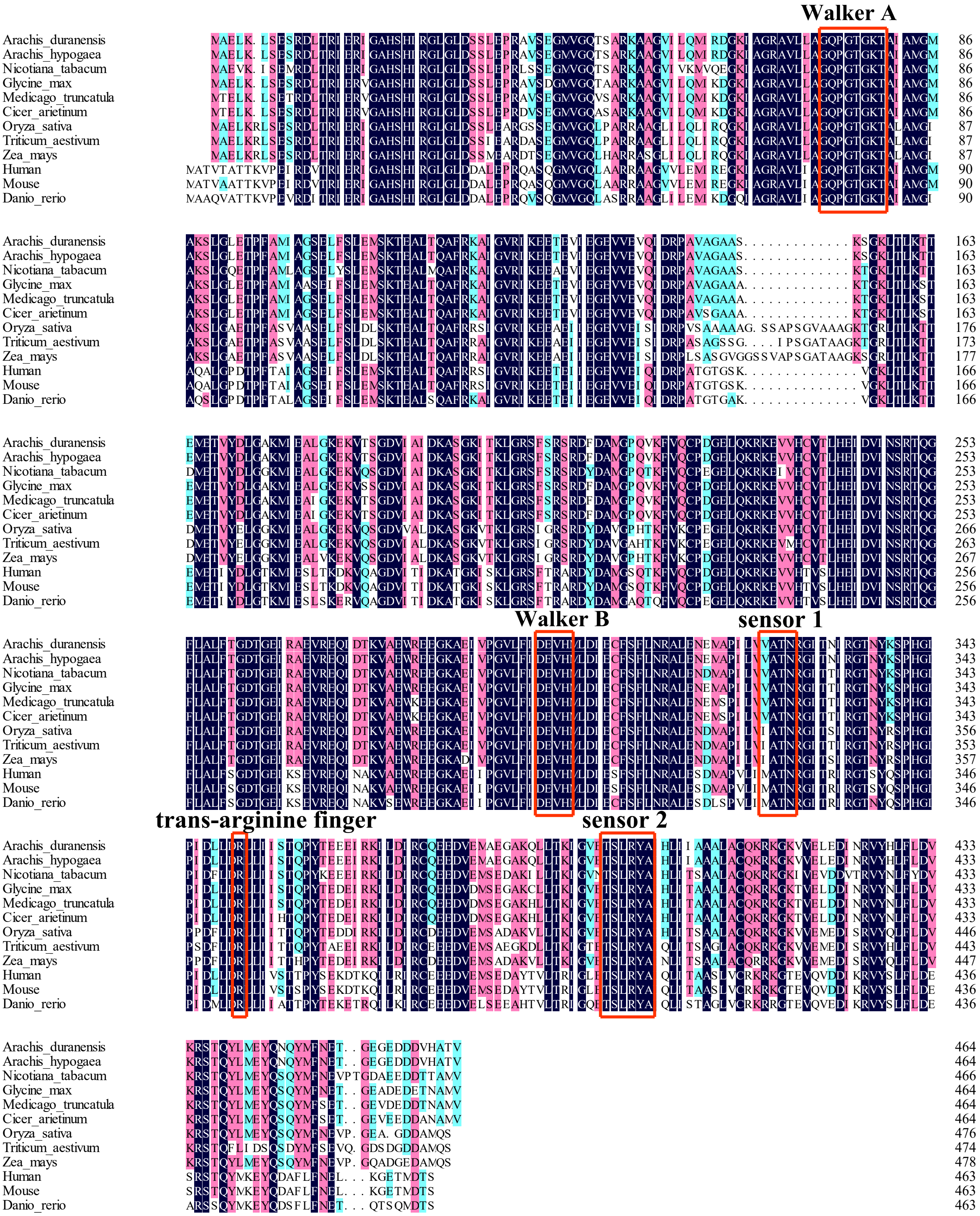

Figure S9 Sequence alignment analyses of RUVBL2 protein in plants and animals . WalkerA, Walker B, sensor 1, sensor 2 and trans-arginine finger in RUVBL2 protein are marked with red box line.
