# Supplementary material for: Fine mapping of qAHPS07 and functional studies of AhRUVBL2 controlling pod size in peanut (Arachis hypogaea L.)
Source: Plant Biotechnol J. 2023 May 31;21(9):1785–98. doi: 10.1111/pbi.14076 (PMC10440995; doi:10.1111/pbi.14076)
Supplement: Supplementary file 10 — Figure S10. Identification of AhRUVBL2 transcription start site by 5'RACE assay in 79266 and D893. [file PBI-21-1785-s008.pdf]

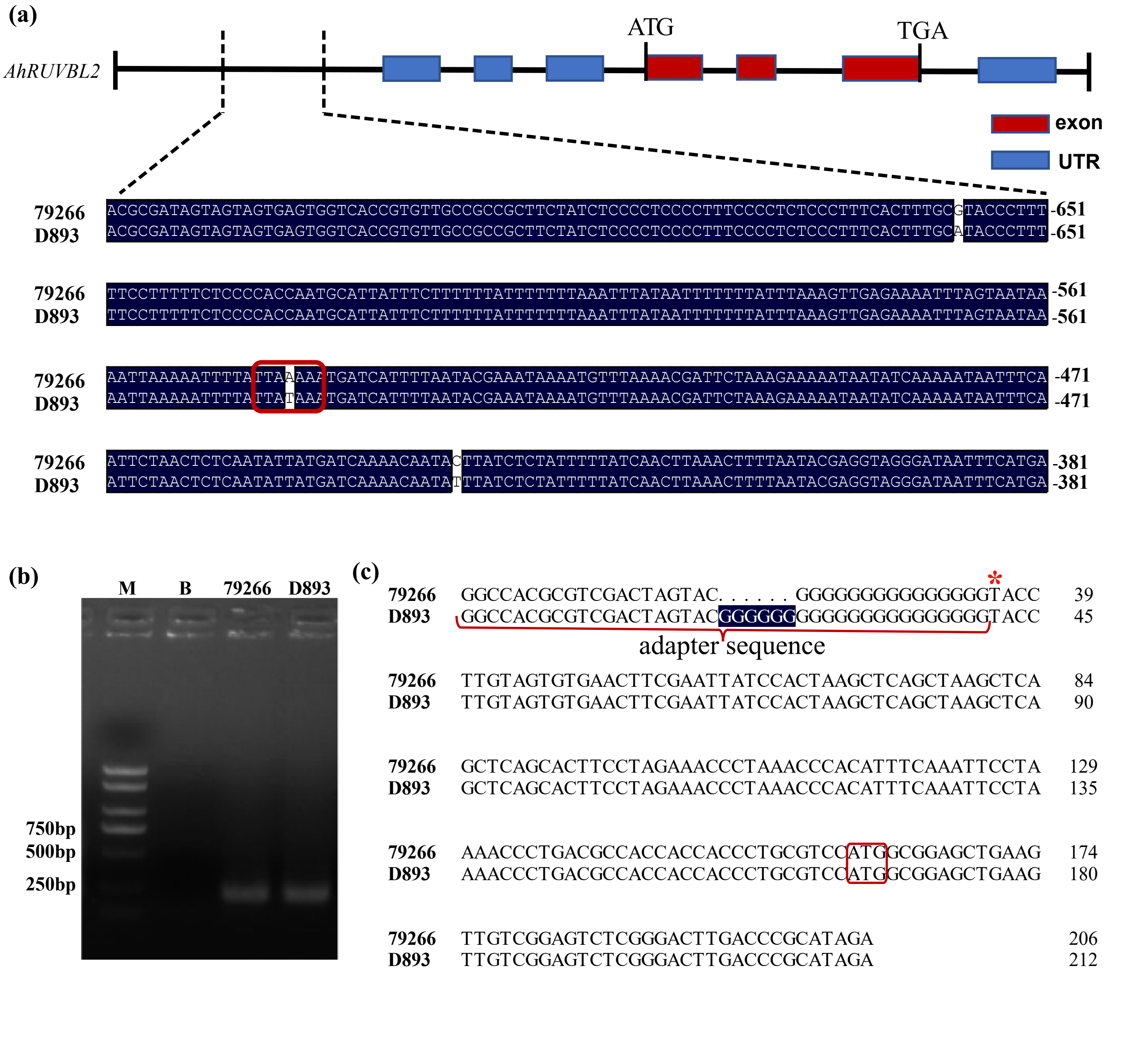

Figure S10.Identication of *AhRUVBL2* transcription start site by 5’ RACE assay in 79266 and D893. (a) Sequence analysis of 79266 and D893 in promoter region of *AhRUVBL2*.(b) 5’ RACE (rapid Amplication of cDNA ends) PCR results. M represents DL2000 marker, B represents blank control.(c) Sequence alignment result. The asterisk “*” represents the transcription start site.
