# Supplementary material for: Fine mapping of qAHPS07 and functional studies of AhRUVBL2 controlling pod size in peanut (Arachis hypogaea L.)
Source: Plant Biotechnol J. 2023 May 31;21(9):1785–98. doi: 10.1111/pbi.14076 (PMC10440995; doi:10.1111/pbi.14076)
Supplement: Supplementary file 11 — Figure S11. Phenotypic analysis of transgenic Arabidopsis. [file PBI-21-1785-s020.pdf]

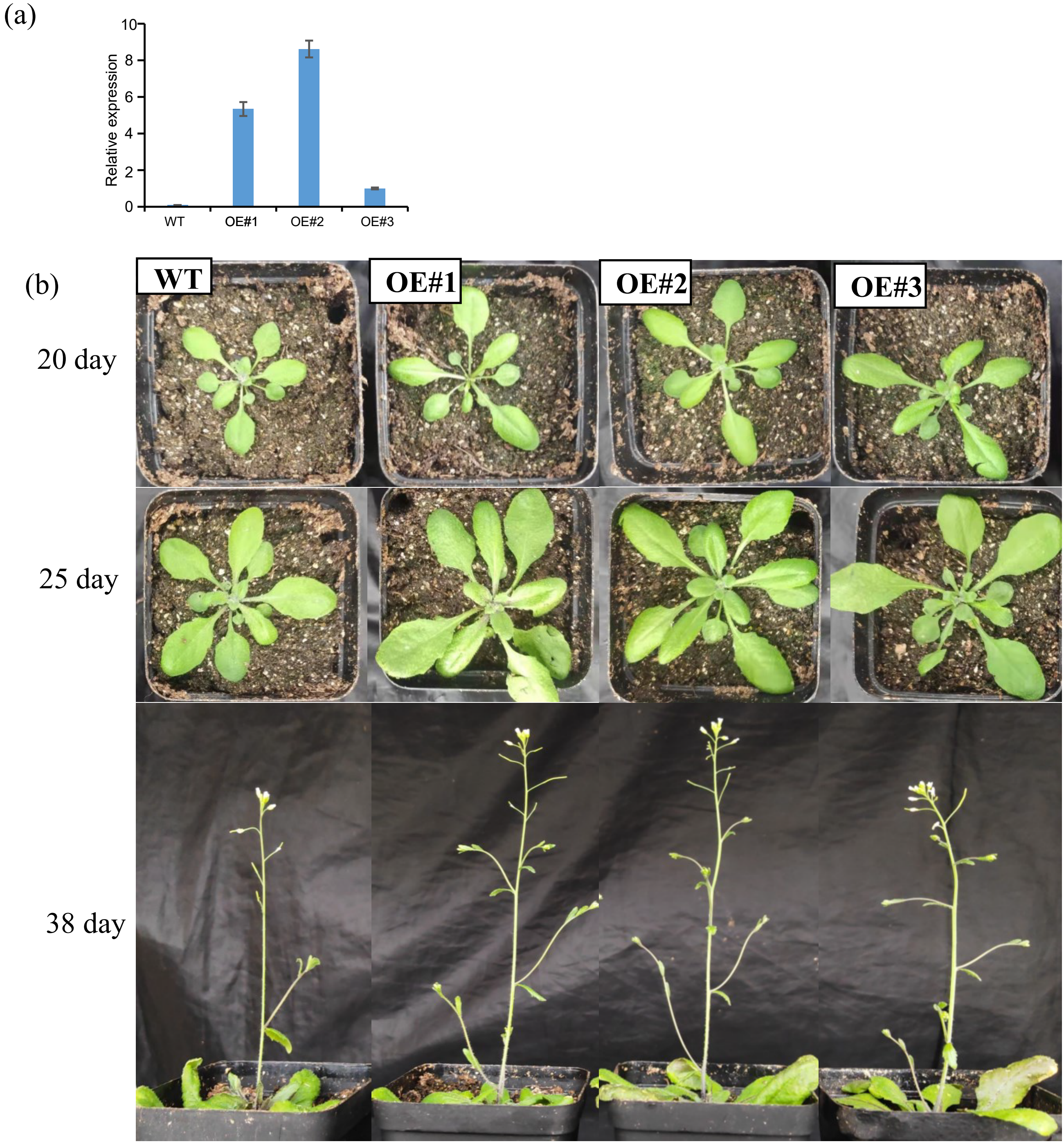

Figure S11 Phenotypic analysis of transgenic *Arabidopsis*.(a)The expression of *AhRUVBL2* in transgenic plants and wild type by qPCR. (b)Transgenic plants and WT plants at 20, 25 and 38 days after germination. WT represents Col-0 *Arabidopsis*; OE#1, OE#2 and OE#3 represent 3 overexpressed transgenic plants.
