# Supplementary material for: Fine mapping of qAHPS07 and functional studies of AhRUVBL2 controlling pod size in peanut (Arachis hypogaea L.)
Source: Plant Biotechnol J. 2023 May 31;21(9):1785–98. doi: 10.1111/pbi.14076 (PMC10440995; doi:10.1111/pbi.14076)
Supplement: Supplementary file 12 — Figure S12. GWAS of single pod weight in 119 cultivated peanut accessions. [file PBI-21-1785-s012.pdf]

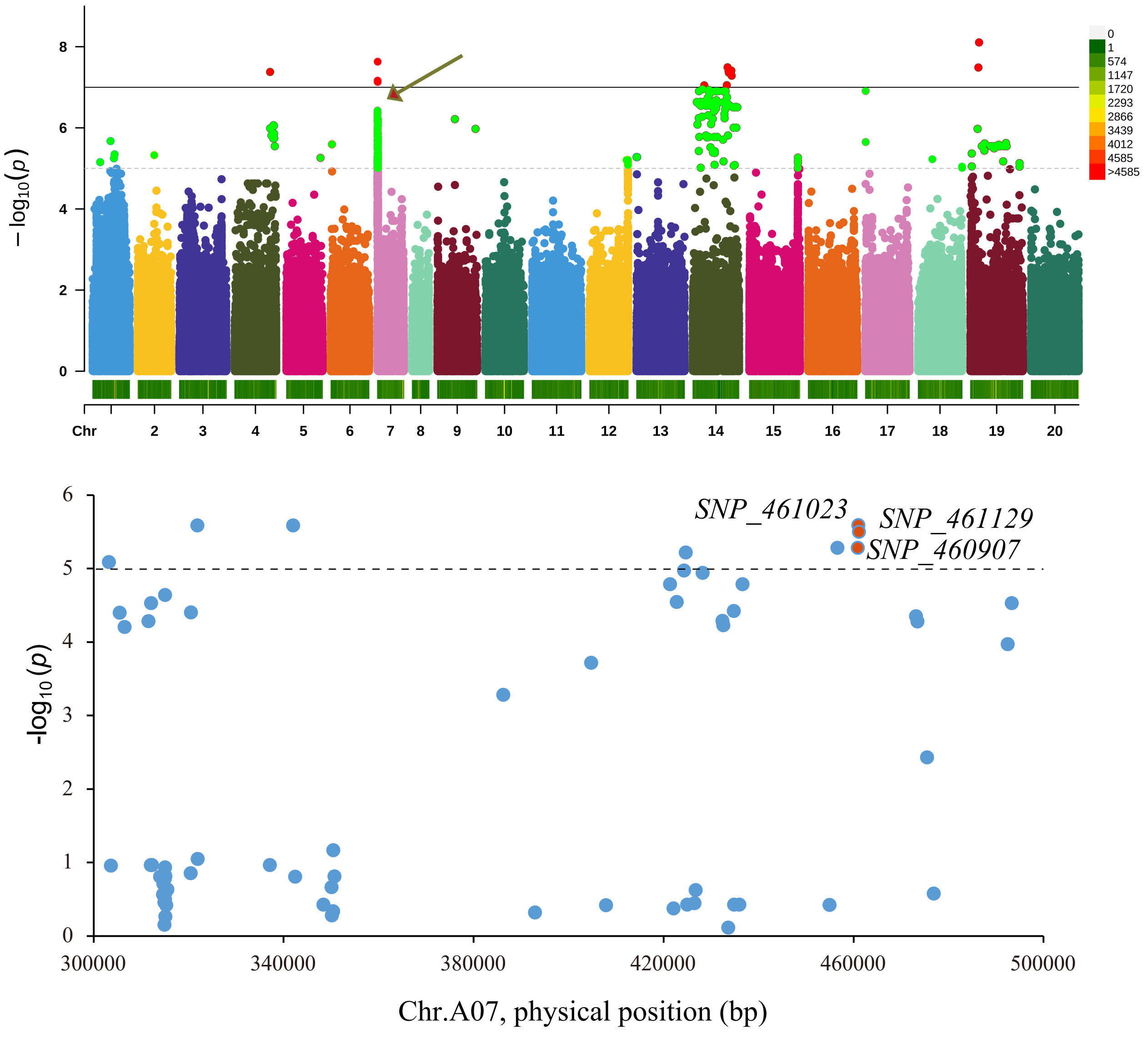

Figure S11 GWAS of single pod weight in 119 cultivated peanut accession. (a) Manhattan plot of GWAS results for single pod weight. (b) Local Manhattan plot for SNPs surrounding the *qAHPS07* on chromosome A07, three SNPs (*SNP_460907*, *SNP_461023* and *SNP_461129*) are shown in red.
