# Supplementary material for: Fine mapping of qAHPS07 and functional studies of AhRUVBL2 controlling pod size in peanut (Arachis hypogaea L.)
Source: Plant Biotechnol J. 2023 May 31;21(9):1785–98. doi: 10.1111/pbi.14076 (PMC10440995; doi:10.1111/pbi.14076)
Supplement: Supplementary file 13 — Table S1. Descriptive statistical analysis of phenotypes of pod‐related traits in the RIL population. [file PBI-21-1785-s018.pdf]

Table S1 Descriptive statistical analysis of phenotypes of pod-related traits in the RIL population

**Traits**	**Planting environment**	**P1**	**P2**	**Min**	**Max**	**Mean**	**SD**	**CV %**	**Sw**	**Kt**
SPW（g）	E1	2.93	3.61**	1.45	3.88	2.53	0.51	20.35	-0.02	-0.42
	E2	3.02	4.12**	1.57	3.8	2.76	0.49	17.92	-0.3	-0.35
	E3	2.42	2.98**	1.33	4.28	2.7	0.62	23.14	-0.14	-0.74
	E4	2.83	3.72**	1.53	4.19	3.01	0.67	22.39	-0.41	-0.83
	E5	2.36	3.32**	1.28	4.44	2.81	0.57	20.45	-0.11	0.03
	E6	2.61	3.58**	1.53	5.05	3.03	0.62	20.58	-0.1	-0.19
	E7	3.38	3.84**	1.74	5.08	3.49	0.68	19.58	-0.11	-0.5
PL（mm）	E1	41.85	50.63**	29.36	49.82	40.6	3.74	9.22	0.04	-0.01
	E2	40.33	50.61**	31.31	49.02	41.45	3.72	8.98	-0.02	-0.56
	E3	38.53	43.93**	31.89	52.32	42.01	4.4	10.47	-0.11	-0.74
	E4	41.15	47.93**	26.95	53.47	43.82	4.74	10.81	-0.26	-0.01
	E5	37.95	46.99**	25.12	58.12	44.52	5.28	11.86	-0.35	0.66
	E6	42.74	49.56**	31.68	57.92	44.41	4.99	11.23	0.06	-0.32
	E7	43.52	53.88**	34.11	61.51	48.7	5.35	10.98	0.07	-0.44
PW（mm）	E1	17.27	20.02**	12.74	20.9	16.83	1.73	10.3	-0.27	-0.26
	E2	16.69	19.99**	11.96	23.32	17.13	1.81	10.57	-0.31	0.78
	E3	15.42	18.72**	12.08	24.63	16.85	2.28	13.52	-0.04	-0.14
	E4	17.8	19.65**	12.37	23.58	17.77	2.44	13.71	-0.25	-0.47
	E5	17.34	20.11**	11.18	22.25	17.51	2.3	13.14	-0.41	-0.23
	E6	16.69	20.12**	12.98	23.5	18	2.4	13.32	-0.17	-0.65
	E7	19.65	20.79**	13.75	27.5	19.41	2.42	12.47	-0.18	-0.04
PST（mm）	E1	1.94	2.66**	1.02	3.98	2.01	0.53	26.58	0.59	0.51
	E2	1.76	2.82**	1.07	3.66	2.28	0.52	22.89	-0.05	-0.28
	E3	1.4	1.86**	0.78	3.09	1.84	0.45	24.74	0.25	-0.47
	E4	1.16	2.59**	0.89	4.16	2.19	0.55	25.21	0.12	0.48
	E5	1.85	2.57**	0.89	3.22	2	0.49	24.28	0.24	-0.35
	E6	1.75	2.35**	1.02	3.18	1.91	0.45	23.39	0.62	-0.04
	E7	2.29	3.24**	1.17	4.27	2.72	0.61	22.61	-0.11	-0.14

P1, 79266; P2, D893; Min, minimum; Max, maximum; SD, standard deviation; CV, coefficient of variation; Sw, skewness; Kt, kurtosis; *Significant at $P<0.05$; **Significant at $P<0.01$.
