# Supplementary material for: Fine mapping of qAHPS07 and functional studies of AhRUVBL2 controlling pod size in peanut (Arachis hypogaea L.)
Source: Plant Biotechnol J. 2023 May 31;21(9):1785–98. doi: 10.1111/pbi.14076 (PMC10440995; doi:10.1111/pbi.14076)
Supplement: Supplementary file 14 — Table S2. Descriptive statistical analysis of phenotypes of pod‐related traits in the F2 population. [file PBI-21-1785-s022.pdf]

Table S2 Descriptive statistical analysis of phenotypes of pod-related traits in the F_2 population

Traits	P1	P2	Min	Max	Mean	SD	CV（%）	Sw	Kt
SPW(g)	2.93	3.61**	0.79	4.25	2.64	0.517	19.54	-0.02	-0.05
PL (mm)	35.33	44.61**	24.9	50.51	41.21	4.177	10.13	-0.44	0.88
PW (mm)	17.27	20.02**	9.47	22.63	17.09	1.54	9.03	0.087	0.75
PST (mm)	1.76	2.82**	0.92	3.92	2.26	0.5	22.39	0.994	6.9

P1, 79266; P2, D893; Min, minimum; Max, maximum; SD, standard deviation; CV, coefficient of variation; Sw, skewness; Kt, kurtosis; *Significant at $P < 0.05$; **Significant at $P < 0.01$.
