# Supplementary material for: Fine mapping of qAHPS07 and functional studies of AhRUVBL2 controlling pod size in peanut (Arachis hypogaea L.)
Source: Plant Biotechnol J. 2023 May 31;21(9):1785–98. doi: 10.1111/pbi.14076 (PMC10440995; doi:10.1111/pbi.14076)
Supplement: Supplementary file 15 — Table S3. Correlation analysis of the pod size‐related traits in F2 population. [file PBI-21-1785-s006.pdf]

Table S3 Correlation analysis of the pod size-related traits in F_2 population

Traits	SPW	PL	PW	PST
SPW	1			
PL	0.59**	1		
PW	0.62**	0.38**	1	
PST	0.44**	0.17	0.49**	1

**Correlation is significant at the 0.01 level.
