# Supplementary material for: Fine mapping of qAHPS07 and functional studies of AhRUVBL2 controlling pod size in peanut (Arachis hypogaea L.)
Source: Plant Biotechnol J. 2023 May 31;21(9):1785–98. doi: 10.1111/pbi.14076 (PMC10440995; doi:10.1111/pbi.14076)
Supplement: Supplementary file 16 — Table S4. Correlation analysis of the pod size‐related traits in RIL population by BLUP. [file PBI-21-1785-s014.pdf]

Table S4 Correlation analysis of the pod size-related traits in RIL population by BLUP

Traits	SPW	PL	PW	PST
SPW	1			
PL	0.754**	1		
PW	0.858**	0.626**	1	
PST	0.733**	0.510**	0.866**	1

**Correlation is significant at the 0.01 level.
