# Supplementary material for: Fine mapping of qAHPS07 and functional studies of AhRUVBL2 controlling pod size in peanut (Arachis hypogaea L.)
Source: Plant Biotechnol J. 2023 May 31;21(9):1785–98. doi: 10.1111/pbi.14076 (PMC10440995; doi:10.1111/pbi.14076)
Supplement: Supplementary file 17 — Table S5. The quality, sequencing depth and coverage of re‐sequencing data of parental lines and bulks. [file PBI-21-1785-s004.pdf]

Table S5 The quality, sequencing depth and coverage of re-sequencing data of parental lines and bulks

**Sample**	**Clean reads**	**Clean bases (G)**	**Genome coverage (%)**	**Mapping ratio (%)**	**Average depth**
79266	701890468	105.28	96.08	99.85	33
D893	553730884	83.05	97.51	99.76	26
Big-bulk	663093616	99.46	98.03	99.72	31
Small-bulk	600487394	90.07	97.94	99.71	27
