# Supplementary material for: Fine mapping of qAHPS07 and functional studies of AhRUVBL2 controlling pod size in peanut (Arachis hypogaea L.)
Source: Plant Biotechnol J. 2023 May 31;21(9):1785–98. doi: 10.1111/pbi.14076 (PMC10440995; doi:10.1111/pbi.14076)
Supplement: Supplementary file 18 — Table S6. KASP markers used in this study. [file PBI-21-1785-s002.pdf]

Table S6 KASP markers used in this study

ID	SNP Allele	SNP Position(bp)	Primer type	Primer sequence
			HEX	GATTCCTCTTCTCAGTCTTTTGTTTC
S7-19	T/C	*Arahy*.07:56535	FAM	AGATTCCTCTTCTCAGTCTTTTGTTTT
			COM	ACGTCAGCTCACTCATCCGCTTAAA
			HEX	ATCTTGCACCATATATATAAAGAGGGC
S7-22	A/C	*Arahy*.07:77772	FAM	GATCTTGCACCATATATATAAAGAGGGA
			COM	CTTGATGATATATGGTGCAATGTACATGTA
			HEX	ACTTAATTGAAAATTTTGCGAAACTATAGAG
S7-38	A/G	*Arahy*.07:142333	FAM	GACTTAATTGAAAATTTTGCGAAACTATAGAA
			COM	GCCTTCCCAACATTTGGGCTCTAAA
			HEX	GCTAAACCACATTAATTCGCCTCATTTT
S7-77	G/A	*Arahy*.07:260733	FAM	CTAAACCACATTAATTCGCCTCATTTC
			COM	CTTGTATGTGTAGCAGGCCCGAATA
			HEX	GTGACTAATGGTGACTATTGGTTGAT
S7-90	G/A	*Arahy*.07:312253	FAM	TGACTAATGGTGACTATTGGTTGAC
			COM	GACATCACTACATACTCCATCTATCACTA
			HEX	ACAGTTATCACCAGAAAGAGTTGAAGA
S7-102	G/A	*Arahy*.07:320503	FAM	CCATGATCTTGATATTCATGCCATTATCTA
			COM	CAGTTATCACCAGAAAGAGTTGAAGG
			HEX	AAGTTCGAGTCTTCCTATTTTTAAAAAAATAT
S7-111	A/T	*Arahy*.07:407879	FAM	AAGTTCGAGTCTTCCTATTTTTAAAAAAATAA
			COM	CATAACTGAAGTTAGTGGTAGCACTCTTT
			HEX	TAATTAGGTTGCGTTTGTTTAGAGAGAT
S7-113	G/A	*Arahy*.07:424670	FAM	TTAGGTTGCGTTTGTTTAGAGAGAC
			COM	CTCTAGATACACTATTTTTATTCATGTCTT
			HEX	ATCAAATTTACATAAAATCGACTCCAGTTA
S7-120	G/A	*Arahy*.07:434824	FAM	TCAAATTTACATAAAATCGACTCCAGTTG
			COM	TGAACTTTGAAGCATTTTGTTTGGTGAAAA
			HEX	CTAACTCTCAATATTATGATCAAAACAATAT
S7-126	C/T	*Arahy*.07:461129	FAM	AACTCTCAATATTATGATCAAAACAATAC
			COM	CCCTACCTCGTATTAAAAGTTTAAGTTGAT
			HEX	ATGGATATATCTAATATTTTATCCTATGGA
S7-130	G/A	*Arahy*.07:504770	FAM	TGGATATATCTAATATTTTATCCTATGGG
			COM	GGGTGAATGTGAATTAGATTAGATTTTGAT
			HEX	TAAATGCAATTCTGTCCATAATACAATAC
S7-132	A/C	*Arahy*.07:524628	FAM	AATAAATGCAATTCTGTCCATAATACAATAA
			COM	CAGAAGCAGGTTTGAAAGTTTGTGTGTTT
			HEX	ACGTGTAAAGGTACTAGTTCTTGATT
S7-150	G/A	*Arahy*.07:606659	FAM	CGTGTAAAGGTACTAGTTCTTGATC
			COM	GTGGTGCCTTCTCTAGACATAATCTATAT
			HEX	ATGCCATATGTTTACGCATGTGCTT
S7-163	C/A	*Arahy*.07:746246	FAM	GCCATATGTTTACGCATGTGCTG
			COM	CAACCATTTGTGACAAAAGTCTTCGGAAT
