# Supplementary material for: Fine mapping of qAHPS07 and functional studies of AhRUVBL2 controlling pod size in peanut (Arachis hypogaea L.)
Source: Plant Biotechnol J. 2023 May 31;21(9):1785–98. doi: 10.1111/pbi.14076 (PMC10440995; doi:10.1111/pbi.14076)
Supplement: Supplementary file 19 — Table S7. QTLs identified for pod size‐related traits in the F2 populations across seven environments. [file PBI-21-1785-s010.pdf]

Table S7 QTLs identified for pod size-related traits in the F_2 populations across seven environments

Trait	QTL	CI (cM)	marker interval	LOD	PVE (%)	Additive
SPW	*FQSPW-1*	0–0.5	*S7-19~S7-22*	24.99	2.39	−0.25
	FQSPW-2	2.5–3.5	*S7-111~S7-126*	4.97	12.36	−0.01
PL	*FQPL*	0–0.5	*S7-19~S7-22*	6.84	4.29	−0.73
PW	*FQPW*	0–1.5	*S7-77~S7-90*	42.79	19.97	−0.96
PST	*FQPST*	2.5–4.5	*S7-111~S7-126*	36.52	16.65	−0.29

CI, Confidence interval of QTLs; LOD, Logarithm of odds; PVE, phenotypic variance explained.
