# Supplementary material for: Fine mapping of qAHPS07 and functional studies of AhRUVBL2 controlling pod size in peanut (Arachis hypogaea L.)
Source: Plant Biotechnol J. 2023 May 31;21(9):1785–98. doi: 10.1111/pbi.14076 (PMC10440995; doi:10.1111/pbi.14076)
Supplement: Supplementary file 20 — Table S8. QTLs identified for pod size‐related traits in the RIL populations across seven environments. [file PBI-21-1785-s017.pdf]

Table S8 QTLs identified for pod size-related traits in the RIL populations across seven environments

Trait	QTL	Environment	CI (cM)	marker interval	LOD	PVE (%)	Additive
SPW	*RQSPW-1*	E5	1.5–3.5	*S7-38~S7-90*	3.83	11.26	−0.19
	RQSPW-2	E1	3.5–4.5	*S7-90~S7-126*	10.34	26.67	−0.26
		E2	3.5–4.5	*S7-90~S7-126*	7.28	21.24	−0.23
		E3	3.5–4.5	*S7-90~S7-126*	19.38	38.6	−0.4
		E4	3.5–4.5	*S7-90~S7-126*	14.58	31.92	−0.39
		E6	3.5–4.5	*S7-90~S7-126*	15.08	34.1	−0.37
		E7	3.5–4.5	*S7-90~S7-126*	47.79	29.78	−0.36
	RQSPW-3	E7	5.5–7	*S7-130~S7-132*	33.83	15.28	−0.29
PL	*RQPL-1*	E1	1.5–3.5	*S7-38~S7-90*	2.62	8.47	−1.08
	RQPL-2	E2	3.5–4.5	*S7-90~S7-126*	3.63	11.33	−1.25
		E3	3.5–4.5	*S7-90~S7-126*	8.64	23.35	−2.12
		E4	3.5–4.5	*S7-90~S7-126*	6.27	17.75	−1.98
		E6	3.5–4.5	*S7-90~S7-126*	5.02	15.14	−1.92
		E7	3.5–4.5	*S7-90~S7-126*	7.2	20.03	−2.38
PW	*RQPW-1*	E5	1.5–3.5	*S7-38~S7-90*	7.31	20.09	−0.97
	RQPW-2	E1	3.5–4.5	*S7-90~S7-126*	11.32	27.96	−0.87
		E2	3.5–4.5	*S7-90~S7-126*	9.94	24.56	−0.86
		E3	3.5–4.5	*S7-90~S7-126*	18.41	34.92	−1.37
		E4	3.5–4.5	*S7-90~S7-126*	13.03	26.7	−1.27
		E6	3.5–4.5	*S7-90~S7-126*	17.55	37.48	−1.45
		E7	3.5–4.5	*S7-90~S7-126*	15.75	33.44	−1.43
PST	*RQPST-1*	E6	0–1.5	*S7-19~S7-38*	7.41	17.57	−0.19
	RQPST-2	E2	1.5–3.5	*S7-38~S7-90*	7.45	17.84	−0.21
	RQPST-3	E5	1.5–3.5	*S7-38~S7-90*	6.16	16.45	−0.19
		E1	3.5–4.5	*S7-90~S7-126*	8.42	20.31	−0.24
		E3	3.5–4.5	*S7-90~S7-126*	9.01	19.07	−0.2
		E4	3.5–4.5	*S7-90~S7-126*	10.56	24.08	−0.26
		E7	3.5–4.5	*S7-90~S7-126*	10.74	25.94	−0.3

The abbreviations used are as in Table S7.
