# Supplementary material for: Fine mapping of qAHPS07 and functional studies of AhRUVBL2 controlling pod size in peanut (Arachis hypogaea L.)
Source: Plant Biotechnol J. 2023 May 31;21(9):1785–98. doi: 10.1111/pbi.14076 (PMC10440995; doi:10.1111/pbi.14076)
Supplement: Supplementary file 21 — Table S9. Information about the six candidate genes and primers were used in qRT‐PCR analysis. [file PBI-21-1785-s001.pdf]

Table S9 Information about the six candidate genes and primers were used in qRT-PCR analysis

**Gene name**	**Gene features**	**Gene Position (bp)**	**Primer name**	**Primer Sequence (5'-3')**
Arahy.5EZV1I	STERILE APETALA-like protein	*Arahy*.07:426329-430608	5E-F	TAACGGAACCAGATTACG
			5E-R	AACTGCTTACAACGAACC
Arahy.LY7S5B	F-box protein	*Arahy*.07:443953-445977	LY-F	ACTACTCCATCCACTCTATCT
			LY-R	CATTCTCCATTCTTCTCCATAC
Arahy.9UY90I	Enolase	*Arahy*.07:447423-452848	9U-F	CACCGAGACCTATCATCAC
			9U-R	TAGTAGCAGCAACATCAAGT
Arahy.RQVC78	uncharacterized protein	*Arahy*.07:447990-448503	RQ-F	TACCACCTGGATATGTTG
			RQ-R	CTATTGTTGGAGTGTTAGAC
Arahy.Y5ZZLQ	neutral/alkaline non-lysosomal ceramidase	*Arahy*.07:453341-458210	Y5-F	CAATAATCCTGCTGAAGAGA
			Y5-R	ATTGAGGTTCCGTGAGTA
Arahy.TSR8I7	RuvB-like2 protein	*Arahy*.07:461567-465885	TS-F	GCCATTCATCTCATCATAGC
			TS-R	CATCGTCGTCTTCACCTT
