# Supplementary material for: Fine mapping of qAHPS07 and functional studies of AhRUVBL2 controlling pod size in peanut (Arachis hypogaea L.)
Source: Plant Biotechnol J. 2023 May 31;21(9):1785–98. doi: 10.1111/pbi.14076 (PMC10440995; doi:10.1111/pbi.14076)
Supplement: Supplementary file 22 — Table S10. Summary of SNPS of parental lines in the candidate interval. [file PBI-21-1785-s021.pdf]

Table S10 Summary of SNPS of parental lines in the candidate interval

**SNP name**	**Position**	**79266 base**	**D893 base**	**SNPs categories**
SNP_424670	*Arahy.07_424670*	G	A	intergenic
SNP_426475	*Arahy.07*_426475	A	C	synonymous
SNP_426760	*Arahy.07*_426760	C	T	intronic
SNP_428205	*Arahy.07*_428205	G	A	intronic
SNP_432409	*Arahy.07*_432409	G	A	intergenic
SNP_432595	*Arahy.07*_432595	C	T	intergenic
SNP_433631	*Arahy.07*_433631	A	G	intergenic
SNP_434824	*Arahy.07*_434824	A	G	intergenic
SNP_435945	*Arahy.07*_435945	T	C	intergenic
SNP_454958	*Arahy.07*_454958	A	G	intronic
SNP_456620	*Arahy.07*_456620	G	A	intronic
SNP_460907	*Arahy.07*_460907	G	A	upstream
SNP_461023	*Arahy.07*_461023	A	T	upstream
SNP_461129	*Arahy.07*_461129	C	T	upstream
