# Supplementary material for: Fine mapping of qAHPS07 and functional studies of AhRUVBL2 controlling pod size in peanut (Arachis hypogaea L.)
Source: Plant Biotechnol J. 2023 May 31;21(9):1785–98. doi: 10.1111/pbi.14076 (PMC10440995; doi:10.1111/pbi.14076)
Supplement: Supplementary file 23 — Table S11. SNPS information in promoter of AhRUVBL2 in 119 cultivated peanut accessions. [file PBI-21-1785-s009.pdf]

Table S11 SNPs information in promoter of *AhRUVBL2* in 119 cultivated peanut accessions

Name	Position(bp)			Haplotype
	***Arahy.*07:460907**	***Arahy.*07:461023**	***Arahy.*07:461129**	
Wendengdalihong	G	A	C	*Hap_GAC*
Laiwupaman	A	T	T	*Hap_ATT*
Yishuidaliman	G	A	C	*Hap_GAC*
Yizhengxiaozi	G	A	C	*Hap_GAC*
Feizhouzhongzhi	G	A	C	*Hap_GAC*
ICG485	G	A	C	*Hap_GAC*
Fozhoujuren	G	A	C	*Hap_GAC*
Sidazhi	A	T	T	*Hap_ATT*
Tongcunzhanyang	G	A	C	*Hap_GAC*
Qianye55	G	A	C	*Hap_GAC*
Qunxuan5	G	A	C	*Hap_GAC*
Linguidazhizi	G	A	C	*Hap_GAC*
Zunhualiyang	G	A	C	*Hap_GAC*
Suiningxiaohuasheng	A	T	T	*Hap_ATT*
Qidongyitouhuasheng	G	A	C	*Hap_GAC*
Lannawan	G	A	C	*Hap_GAC*
Zhaoyuanbanman	A	T	T	*Hap_ATT*
Penglaiyiwohou	G	A	C	*Hap_GAC*
Junansongkangxianchong	G	A	C	*Hap_GAC*
NC6	G	A	C	*Hap_GAC*
Hua17	G	A	C	*Hap_GAC*
NC4	G	A	C	*Hap_GAC*
Zhouye	G	A	C	*Hap_GAC*
ICG446	G	A	C	*Hap_GAC*
Fozhoumansheng	A	T	T	*Hap_ATT*
M13	G	A	C	*Hap_GAC*
Makulu red	G	A	C	*Hap_GAC*
Jinkins Jumbo	G	A	T	*Hap_GAT*
Altiko	G	A	T	*Hap_GAT*
Dongpingdapaman	A	T	T	*Hap_ATT*
Shijiazhuanghuasheng	G	A	C	*Hap_GAC*
Funingduoli	G	A	C	*Hap_GAC*
Qinhuangdaoliyang	A	T	T	*Hap_ATT*
Rizhaodaguozi	G	A	C	*Hap_GAC*
Jinzhou171-6-3	A	T	T	*Hap_ATT*
Qinglan2	G	A	C	*Hap_GAC*
Nadadadou	G	A	C	*Hap_GAC*
Jihua2	G	A	C	*Hap_GAC*
Bujieliuxi	G	A	C	*Hap_GAC*
Nongda818	G	A	C	*Hap_GAC*

Name	Position(bp)			Haplotype
	Arahy.07:460907	*Arahy*.07:461023	*Arahy*.07:461129	
Hua17xiaoguo	G	A	C	*Hap_GAC*
Early bunch	G	A	C	*Hap_GAC*
ICGV86699	G	A	C	*Hap_GAC*
Ehua3	A	T	T	*Hap_ATT*
Ehua2	A	T	T	*Hap_ATT*
Yuhua1	G	A	C	*Hap_GAC*
Luhua1	A	T	T	*Hap_ATT*
Linhua1	A	T	T	*Hap_ATT*
Shanhua17	G	A	C	*Hap_GAC*
02H061	A	T	T	*Hap_ATT*
Ji0608-4-9	G	A	C	*Hap_GAC*
Ji0607-17	G	A	C	*Hap_GAC*
Ji9402	G	A	C	*Hap_GAC*
Tedaguo（Bai╳Peng）	A	T	T	*Hap_ATT*
Yeza1	G	A	C	*Hap_GAC*
98D080-2	G	A	C	*Hap_GAC*
98H269	G	A	C	*Hap_GAC*
97H066	G	A	C	*Hap_GAC*
97H017	A	T	T	*Hap_ATT*
97H027	G	A	C	*Hap_GAC*
Fenghua5	G	A	C	*Hap_GAC*
03F032	A	T	T	*Hap_ATT*
Kangqing19	A	T	T	*Hap_ATT*
Junan2	A	T	T	*Hap_ATT*
02D431	G	A	C	*Hap_GAC*
Fenghua3	G	A	C	*Hap_GAC*
02P186	A	T	T	*Hap_ATT*
Jihua5	G	A	C	*Hap_GAC*
04D029	A	T	T	*Hap_ATT*
F20/DQ1011	G	A	C	*Hap_GAC*
P10-8/DQ1112	G	A	C	*Hap_GAC*
F18/WQ1102	G	A	C	*Hap_GAC*
DQ1212	G	A	C	*Hap_GAC*
Nongda831	G	A	C	*Hap_GAC*
07H266	A	T	T	*Hap_ATT*
Jihua8	G	A	C	*Hap_GAC*
Fenhua6	G	A	C	*Hap_GAC*
Fenghua1	A	T	T	*Hap_ATT*
Shanhua7	G	A	C	*Hap_GAC*
Shanhua9	A	T	T	*Hap_ATT*
Shanhua11	G	A	C	*Hap_GAC*
Huayu22	G	A	C	*Hap_GAC*

Name	Position(bp)			Haplotype
	***Arahy.*07:460907**	***Arahy.*07:461023**	***Arahy.*07:461129**	
Luhua11	G	A	C	*Hap_GAC*
Xuzhou68-4	A	T	T	*Hap_ATT*
Huayu25	A	T	T	*Hap_ATT*
Yuhua15	G	A	C	*Hap_GAC*
Huayu17	G	A	C	*Hap_GAC*
Luhua8	A	T	T	*Hap_ATT*
Luhua14	A	T	T	*Hap_ATT*
Ji0212-4	G	A	C	*Hap_GAC*
Xianghua2008	A	T	T	*Hap_ATT*
Weihua10	G	A	C	*Hap_GAC*
Weihua11	G	A	C	*Hap_GAC*
Huayu31	G	A	C	*Hap_GAC*
Haihua1	A	T	T	*Hap_ATT*
Shanhua15	A	T	T	*Hap_ATT*
98H013	A	T	T	*Hap_ATT*
Erlihei	A	T	T	*Hap_ATT*
Dalimeiguihong	A	T	T	*Hap_ATT*
Kainong8	G	A	C	*Hap_GAC*
Xinjianshehuasheng	G	A	C	*Hap_GAC*
Sihongdahuasheng	G	A	C	*Hap_GAC*
51015-2	A	T	T	*Hap_ATT*
Wenanfenghuangwo	G	A	C	*Hap_GAC*
83-13	A	T	T	*Hap_ATT*
Meiyinxuan41159	G	A	C	*Hap_GAC*
Haiyangdalidun	G	A	C	*Hap_GAC*
Qingyuanyiwohou	A	T	T	*Hap_ATT*
1977/6/4	A	T	T	*Hap_ATT*
6622	G	A	C	*Hap_GAC*
VA850611	A	T	T	*Hap_ATT*
PI162857	G	A	C	*Hap_GAC*
PI295250	G	A	C	*Hap_GAC*
PI295309	G	A	C	*Hap_GAC*
PI468250	G	A	C	*Hap_GAC*
PI323268	G	A	C	*Hap_GAC*
PI504614	G	A	T	*Hap_GAT*
05H511	G	A	C	*Hap_GAC*
Juhua27	G	A	C	*Hap_GAC*
