# Supplementary material for: Fine mapping of qAHPS07 and functional studies of AhRUVBL2 controlling pod size in peanut (Arachis hypogaea L.)
Source: Plant Biotechnol J. 2023 May 31;21(9):1785–98. doi: 10.1111/pbi.14076 (PMC10440995; doi:10.1111/pbi.14076)
Supplement: Supplementary file 24 — Table S12. Genespecific primers for AhRUVBL2. [file PBI-21-1785-s016.pdf]

Table S12 Gene specific primers for *AhRUVBL2*

Primer name	Sequence （5' to 3'）	Description
AhRUVBL2-F	ATGGCGGAGCTGAAGTTGTCGG	Forward primer of CDS region of *AhRUVBL2*
AhRUVBL2-R	TTAAAAGACCGTGGCATGGACATCG	Reverse primer of CDS region of *AhRUVBL2*
TAhRUVBL2-F	GATCTGCTTGATCGTCTGC	Forward primer for pTRV2:*AhRUVBL2*
TAhRUVBL2-R	CTGATTCTGATACTTTAT	Reverse primer for pTRV2:*AhRUVBL2*
GSP1	GCGAGGCTCGAGAGAA	*GSP1,GSP2* and *GSP3* used for the localization of the transcriptional start site of *AhRUVBL2* gene
GSP2	CGAATGTGAGAGTGAGCG	
GSP3	CTCTATGCGGGTCAAGTC	
